# Serologic and urinary characteristics of laboratory-confirmed genitourinary tuberculosis at a tertiary hospital in the Philippines

**DOI:** 10.1186/s12894-021-00888-3

**Published:** 2021-09-09

**Authors:** Paolo Nikolai H. So, Anthony Russell T. Villanueva

**Affiliations:** Division of Nephrology, Department of Medicine, University of the Philippines Manila – Philippine General Hospital, Taft Avenue, Ermita, 1000 Manila, Philippines

**Keywords:** Genitourinary tuberculosis, Electrolytes, Urinalysis, Association

## Abstract

**Background:**

Genitourinary tuberculosis (GUTB) is known to cause high rates of structural organ damage, however, literature on its biochemical manifestations is limited. Additionally, local studies in the Philippine setting, where cases are rampant, are few and dated. This study aimed to determine the serologic and urinary profile of patients with GUTB admitted at a tertiary hospital within January 2009 to March 2020 and their association with short-term outcomes.

**Methods:**

This retrospective study included 112 patients with laboratory-confirmed GUTB (i.e., positivity in acid-fast smear, polymerase chain reaction, culture, or histology). Demographic data, clinical characteristics, laboratory and radiologic findings, histopathology reports, treatment, and short-term outcomes were recorded.

**Results:**

Bladder (54.5%) and kidney (36.4%) were the most affected organs. The male:female ratio was 1:1.15, and the mean age was 35.79 ± 18.29 years. Weakness (14.29%) was the most common chief complaint. A majority presented with anemia (83.04%), while several had leukocytosis (41.96%) and thrombocytosis (26.79%). Hypoalbuminemia (58.10%), impairment of renal function (36.94%), and electrolyte abnormalities such as hyponatremia (50.93%), hypercalcemia (20.19%), and hypokalemia (21.82%) were common. Proteinuria (67.96%) and pyuria (67.96%) were the most frequent abnormal findings, followed by hematuria (51.46%), acidic urine (45.63%) and low specific gravity (31.07%). Age, leukocytosis, and the need for pressors were all significantly associated with mortality (*p* values of <0.001, 0.010, and <0.001, respectively).

**Conclusions:**

The young age at presentation with severe clinical and laboratory manifestations may reflect local epidemiology as TB continues to be widespread in the country. Apart from the more commonly cited abnormalities in literature, multiple electrolyte imbalances and urinary concentration defects were also observed in many cases, possibly indicating tubulointerstitial involvement—a complication increasingly mentioned in case reports. As several patient characteristics were found to be associated with the high mortality rates observed in the study, further research is recommended to explore predictive modeling.

## Introduction

Tuberculosis (TB) remains an important global epidemic, with latest estimates of disease burden amounting to 10.0 (range, 9.0–11.1) million people in 2018 [1]. Extrapulmonary TB (EPTB) is reported to comprise around 16.5–25% of all cases, attributing 4.5–27% to genitourinary TB (GUTB) [2–4]. GUTB historically pertains to the infection of the urogenital system organs in any combination by *Mycobacterium tuberculosis* (MTB) or *Mycobacterium bovis* [5–8]. It presents with insidious and late-onset symptoms, making its diagnosis and treatment difficult and delayed, and consequently leading to high rates of structural organ damage and kidney failure [9, 10]. Some physicians advocate the term *urogenital TB* (UGTB) as kidney TB is the most relevant infection and is more frequently diagnosed than genital TB [5, 6, 11].

Philippines ranked 4th among the countries with high TB burden in 2018, accounting 6% of the global total. It has a TB incidence rate of 554 (311–866) per 100,000 population, and a mortality rate of 24.57 (20–32) per 100,000 population [1]. This high disease prevalence in the country is multifactorial, attributing to high poverty rate, marked social inequities, and rise in slum housing and crowded living conditions from rapid urbanization [12]. In the Filipino pediatric population, GUTB is found to cause 3% of extrapulmonary TB cases admitted in a tertiary hospital [13].

Despite these figures, there is paucity in literature regarding local experience on GUTB, especially with regards to serologic and urinary findings [14–16]. The last available reference was published 25 years ago, which also needs to be updated. The study aims to determine the serologic and urinary profile of patients with genitourinary TB admitted at a tertiary government hospital in Philippines and their association with short-term outcomes.

## Methods

### Study design and population

This is a single-center, retrospective study performed at the Philippine General Hospital (PGH; 1500 beds). This study included GUTB patients diagnosed from January 2009 to March 2020 through positivity in at least one of the following: (1) urine acid-fast bacilli (AFB) staining, (2) urine or tissue polymerase chain reaction (PCR) for *Mycobacterium tuberculosis*, (3) urine or tissue *M. tuberculosis* culture, and (4) histologic findings of granulomatous inflammation (granulomas composed of epithelioid cells and Langhans giant cells with or without caseous necrosis) [14, 15, 17–19]. Culture-positive samples solely involving the female genital tract without urologic involvement, out-patients, in-patients without baseline serologic and urinary tests, and those who got discharged against medical advice were excluded. This study was approved by the University of the Philippines Manila Research Ethics Board and the requirement for informed consent was waived since the investigators evaluated anonymized data.

### Data collection and definition of variables

Data collection was done through chart review of patient medical records. This included patient demographics; comorbidities; clinical symptoms; complete blood counts; serum chemistries; urinalysis; results of microbial smears and cultures, diagnostics, and histopathology; treatment; and short-term outcomes. Organ involvement was distinguished in those with tissue samples obtained from biopsies or operations.

Serologic abnormalities were defined as follows: (1) anemia as hemoglobin of < 150 g/L in neonates 0–30 days old, < 105 g/L in 1–23 months of age, < 115 g/L in children 2–9 years of age, < 125 g/L in males 10–17 years of age, < 120 g/L in non-pregnant females 10 years of age and above, < 110 g/L in pregnant women, and < 130 g/L in men 15 years of age and above [20, 21]; (2) thrombocytopenia as platelets < 84,000/µL in newborns ≤ 1 week old, and < 150,000/µL for the rest of the age groups [21, 22]; (3) thrombocytosis as platelets > 450,000/µL [22]; (4) leukocytosis as white blood cells > 34,000/µL in neonates 0–30 days, > 14,000/µL in infants 1–23 months of age, > 12,000/µL in 2–9 years of age, > 10,500/µL in 10–18 years of age, and > 11,000/µL in adults [21, 23]; (5) leukopenia as white blood cells < 9100/µL in neonates 0–30 days, < 6000/µL in infants 1–23 months of age, < 4000/µL in 2–18 years of age, and < 4400/µL in adults [21, 23]; (6) hypoalbuminemia as serum albumin < 18 g/L in premature neonates 1 day old, < 25 g/L in full term neonates < 6 days old, < 19 g/L in 8 days-1 year old, < 34 g/L in 1–3 years of age, < 35 g/L in 4–19 years of age, and < 34 g/L in adults [21]; (7) impaired renal function as estimated glomerular filtration rate < 60 mL/min/1.73 m [2]; (8) hyperkalemia as plasma K^+^ concentration≥ 5.5 mM [22], (2) hypokalemia as plasma K^+^ concentration < 3.5 mM [22], (9) hyponatremia as plasma Na^+^ concentration < 135 mM [22], and (10) hypercalcemia as total serum calcium concentration ≥ 10.4 mg/dL [24].

Urinary findings were defined as: (1) acidic urine as urine pH ≤ 5.5 [24], (2) low specific gravity as urine specific gravity ≤ 1.010, (3) hematuria as three or more erythrocytes per high-power field [24], (4) proteinuria as detection of proteinuria by dipstick examination [22], and (5) pyuria as detection of more than 5 white blood cells per high-power field in urine microscopy or positive leukocyte esterase dipstick testing [15, 24].

### Statistical analysis

Descriptive statistics were used in the analysis of this study. Frequency and percentage were used to describe categorical variables and proportions of patients who improved, expired, or developed short-term outcomes such as the need for pressors or renal replacement therapy. Continuous variables were expressed as median.

Associations were determined by bivariate analysis using Fisher’s exact test for characteristics involving 2 categories or Chi-square test for those with > 2 categories. Mean lengths of hospital stay between those with and without the identified clinical, serologic, and urinary characteristics were compared using Mann–Whitney U-test for characteristics involving 2 categories or Kruskal–Wallis test for those with > 2 categories. For all tests, *p* value of at most 0.05 indicate significance.

## Results

**Fig. 1:**
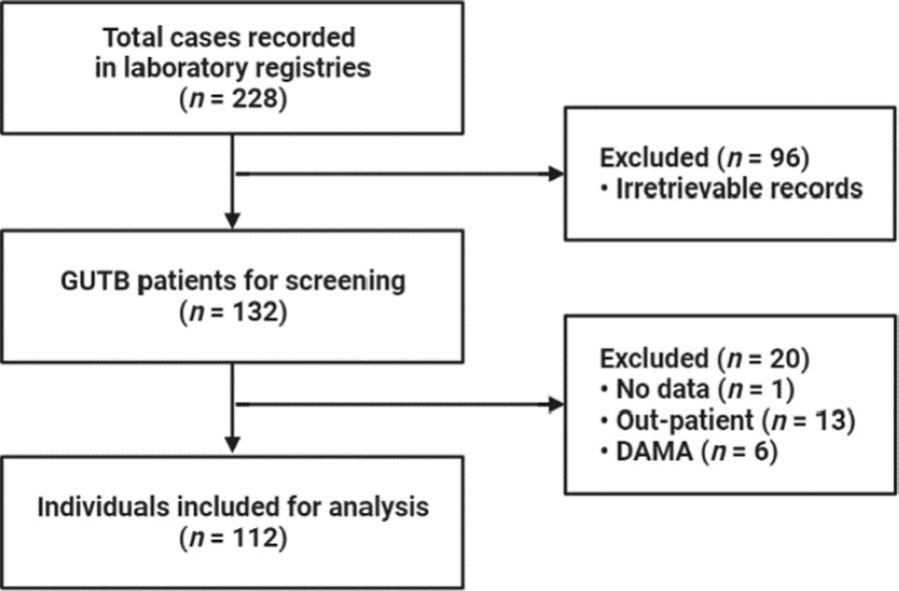
Flow diagram of study selection. *DAMA* discharged against medical advice

A total of 228 patients with laboratory-confirmed GUTB were identified. Ninety-six charts were irretrievable due to institutional limitations in records retention, while 20 cases met the exclusion criteria (Fig. 1). Among the 112 patients included in the study, half (50.0%) had positive smears for urine AFB (Table 1). In those with histopathologic evidence of infection, bladder (n = 12, 54.5%) and kidney (n = 8, 36.4%) were the most involved genitourinary organs. Among those with kidney and ureter involvement, left laterality was observed in 60% (Table 2).

**Table 1:** Proportion of patients with GUTB

Diagnosis	No. of patients (%) (n = 112)
Urine AFB smear-positive	56 (50.00%)
Urine PCR-positive	16 (14.29%)
Urine culture-confirmed	18 (16.07%)
Histopathology	22 (19.64%)
Bladder	12
Kidney	6
Kidney and ureter	2
Ureter	2

**Table 2:** Laterality of organ involvement in GUTB patients confirmed by histopathology

Organ	No. of patients (%) (n = 10)		
	Right	Left	Unspecified
Kidney	1	4	1
Kidney and ureter	0	2	0
Ureter	1	0	1
Total	2 (20.0%)	6 (60.0%)	2 (20.0%)

### Patient characteristics

Baseline clinical characteristics of the patients with GUTB are shown in Table 3. The mean age (± SD) was 35.79 ± 18.29 years (range, 1–82 years) and the male-to-female ratio was 1:1.15 (52:60). Most patients were single or widowed (61.61%) and lived in urban areas (53.57%). Fourteen patients (12.5%) had a previous history of tuberculosis, while 64 patients (57.14%) had present evidence of other organ involvement, with lungs (50.89%) being the most concomitantly involved organ. Twenty-four patients (21.43%) exhibited systemic symptoms such as weakness (14.29%) and fever (7.14%), while 57 patients (50.89%) had genitourinary manifestations as their chief complaint. Flank or abdominal pain was the most common presenting genitourinary symptom (21.43%).

**Table 3:** Clinical characteristics of patients with GUTB

Clinical characteristics	No. of patients (%) (n = 112)
Gender	
M	52 (46.43%)
F	60 (53.57%)
Age	
0 months–1 year	1 (0.89%)
1 year–5 years	2 (1.79%)
6 years–10 years	4 (3.57%)
11 years–18 years	18 (16.07%)
19 years–29 years	21 (18.75%)
30 years–49 years	35 (31.25%)
50 years–69 years	27 (24.11%)
70 years or older	4 (3.57%)
Marital status	
Single/widowed	69 (61.61%)
Married	43 (38.39%)
Occupation	
Employed	19 (16.96%)
Unemployed	58 (51.79%)
Not applicable (i.e., pediatric)	26 (23.21%)
Unspecified	9 (8.04%)
Location	
Urban	60 (53.57%)
Rural	48 (42.86%)
Unspecified	4 (3.57%)
*Co-morbidity*	
Previous TB	14 (12.50%)
Diabetes mellitus	5 (4.46%)
HIV/AIDS	12 (10.71%)
Steroid use (e.g., SLE, NS)	9 (8.04%)
Malignancy	1 (0.89%)
Chronic kidney disease	5 (4.46%)
History of urolithiasis	8 (7.14%)
RTA Type 1	1 (0.89%)
COPD	2 (1.79%)
Bronchial asthma	2 (1.79%)
Hypertension	13 (11.61%)
Heart failure	2 (1.79%)
Cerebrovascular disease	1 (0.89%)
*Other organ involvement*	64 (57.14%)
Pulmonary	57 (50.89%)
Gastrointestinal	28 (25.00%)
Abdominopelvic	11 (9.82%)
Central nervous system	7 (6.25%)
Bone	4 (3.57%)
Lymph node	8 (7.14%)
Ear	2 (1.79%)
Psoas	2 (1.79%)
Cutaneous/wound	3 (2.68%)
*Chief complaint*	
Weakness	16 (14.29%)
Difficulty of breathing	14 (12.50%)
Flank pain	13 (11.61%)
Abdominal pain	11 (9.82%)
Hematuria	9 (8.04%)
Dysuria	9 (8.04%)
Fever	8 (7.14%)
Abdominal/pelvic mass on diagnostic	5 (4.46%)
Pedal edema	4 (3.57%)
Umbilical discharge	3 (2.68%)
Gluteal pain	2 (1.79%)
Vaginal bleeding	2 (1.79%)
Cough	2 (1.79%)
Seizure	2 (1.79%)
Urinary retention	1 (0.89%)
Inguinal pain	1 (0.89%)
Fistula formation (ureterocutaneous)	1 (0.89%)
Scrotal discharge	1 (0.89%)
Double J stent reinsertion	1 (0.89%)
Decrease in sensorium	1 (0.89%)
Vomiting	1 (0.89%)
Others	5 (4.46%)

*COPD* chronic obstructive pulmonary disease, *F* female, *HIV/AIDS* human immunodeficiency virus or acquired immunodeficiency syndrome, *M* male, *NS* nephrotic syndrome, *RTA Type 1* renal tubular acidosis Type 1, *SLE* systemic lupus erythematosus, *TB* tuberculosis

### Serologic and urinary characteristics

Majority of patients presented with anemia (83.04%), while several exhibited leukocytosis (41.96%) and thrombocytosis (26.79%) (Table 4). Of the biochemistry data, hypoalbuminemia (58.10%) was the most common, followed by hyponatremia (50.93%), impairment of renal function (36.94%), hypercalcemia (20.19%), and hypokalemia (21.82%). Of those with urine samples, proteinuria (67.96%) and pyuria (67.96%) were the most common abnormal findings, followed by hematuria (51.46%), acidic urine (45.63%), and low specific gravity (31.07%) (Table 5).

**Table 4:** Serologic characteristics of patients with GUTB

Serologic abnormalities	No. of patients (%)
Anemia	93 (83.04%, n = 112)
Thrombocytopenia	6 (5.36%, n = 112)
Thrombocytosis	30 (26.79%, n = 112)
Leukocytosis	47 (41.96%, n = 112)
Leukopenia	6 (5.36%, n = 112)
Hypoalbuminemia	61 (58.10%, n = 105)
Renal function impairment	41 (36.94%, n = 111)
Hyperkalemia	9 (8.18%, n = 110)
Hypokalemia	24 (21.82%, n = 110)
Hyponatremia	55 (50.93%, n = 108)
Hypercalcemia	21 (20.19%, n = 104)

**Table 5:** Urinary characteristics of patients with GUTB

Urinary abnormalities	No. of patients (%) (n = 103)
Acidic pH	47 (45.63%)
Low specific gravity	32 (31.07%)
Proteinuria	70 (67.96%)
Negative	33 (32.04%)
Trace	15 (14.56%)
1+	36 (34.95%)
2+	16 (15.53%)
3+	3 (2.91%)
Hematuria	53 (51.46%)
Pyuria	70 (67.96%)
Pyuria + hematuria	50 (48.54%)
Casts	17 (16.50%)
Crystals	4 (3.88%)

### Radiological findings

Two patients underwent intravenous pyelography (IVP), with one showing extensive calcifications throughout the urinary tract, while the other revealing non-functioning kidney. Table 6 shows the rest of the imaging findings observed in our investigation.

**Table 6:** Imaging findings associated with GUTB

*Intravenous pyelography*
Unilateral renal parenchymal disease Non-functioning kidney Calcification of the urinary tract: medullary nephrocalcinosis, nephrolithiasis, ureterolithiasis, cystolithiasis Bladder wall thickening
*CT scan*
Hypodense renal foci with or without internal septations or peripheral calcifications Renal cysts, Bosniak I and II Renal mass Calcification of the urinary tract: nephrolithiasis, ureteropelvic junction lithiases Urinary tract dilatation: hydronephrosis, ureteropelvocaliectasia with possible distal ureteral stricture Ureteral wall thickening Bladder wall thickening Vesicocutaneous fistulous tract Evidence of extra-renal TB infection: Pulmonary tuberculosis with or without endobrochial spread Distal ileal and ileocecal wall thickening with multiple abscess formation (intraabdominal, pelvic, and prostatic regions) and lymphadenopathies Multilevel vertebral lesions with disc destruction (Pott’s disease) with abscess formation involving adjacent muscles (psoas, iliopsoas and gluteus maximus)
*Ultrasound*
Unilateral or bilateral renal parenchymal disease with or without signs of chronicity Echogenic renal walls with or without internal echoes suggestive of pyelitis or pyelonephritis Pyonephrosis Renal cysts or mass Calcification of the urinary tract: nephrocalcinosis, nonspecific parenchymal/perinephric/periureteral calcifications, nephrolithiasis, urolithiases, Urinary tract dilatation: hydronephrosis, focal caliectasia, pelvocaliectasia, ureteropelvocaliectasia Irregular, diffuse, or heterogeneous bladder wall thickening Bladder wall foci or mass Evidence of abdominopelvic Koch’s infection: tobacco pouch appearance of fallopian tube, thickening of uterine serosa and peritoneum, palisading bowel loops, and massive ascites

### Treatment

Seventy-one patients (63.39%) were initiated on anti-Koch’s treatment during their admission, while 29 individuals (25.89%) underwent surgery. Double J stent insertion (9.82%) and percutaneous tube nephrostomy (8.93%) were the most performed urologic operations (Table 7).

**Table 7:** Management of admitted patients with GUTB

Intervention	No. of patients (%) (n = 112)
Anti-Koch’s therapy	71 (63.39%)
Operation	29 (25.89%)
Percutaneous tube nephrostomy	10 (8.93%)
Double J stent insertion	11 (9.82%)
Nephrectomy	2 (1.79%)
Subcapsular nephrectomy	5 (4.46%)
Cytoreductive nephrectomy	1 (0.89%)
Aspiration of renal abscess	1 (0.89%)
Radial nephrolithotomy	1 (0.89%)
Pelvolithotomy	1 (0.89%)
Ureterotomy	1 (0.89%)
Ureteroneocystostomy	1 (0.89%)
Bladder mass excision	3 (2.68%)
Transurethral resection of bladder tumor	1 (0.89%)

### Short-term outcomes

In-hospital death occurred in 8.04% of the patients. The median hospital length of stay was 11 days, with a minimum hospital stay of 0.67 day to a maximum of 90 days. Fifteen patients (13.39%) required pressors and 1 patient (0.89%) needed renal replacement therapy in the form of hemodialysis throughout their hospital course (Table 8).

**Table 8:** Short-term outcomes of admitted patients with GUTB

Length of hospital stay in days, median (min–max)	11 (0.67–90)
Improved, n (%)	103 (91.96%)
Expired, n (%)	9 (8.04%)
Need for pressors, n (%)	15 (13.39%)
Need for renal replacement therapy, n (%)	1 (0.89%)

### Characteristics associated with short-term outcomes

#### Mortality

Age, leukocytosis, and the need for pressors were all significantly associated with mortality (*p* values of <0.001, 0.010, and <0.001, respectively) (Tables 9, 10, 13). Other characteristics were not significantly associated with mortality (Tables 9, 10, 11, 12, 13).

**Table 9:** Bivariate analysis: clinical characteristics and mortality

Characteristic	Category	Mortality				*p* value
		Survived		Expired		
		Count	Row %	Count	Row %	
Gender	Male	48	92.3	4	7.7	1.000
	Female	55	91.7	5	8.3	
Age	0 months to 1 year	0	0.0	1	100.0	**<0.001**
	1–5 years	0	0.0	2	100.0	
	6–10 years	4	100.0	0	0.0	
	11–18 years	17	94.4	1	5.6	
	19–29 years	19	90.5	2	9.5	
	30–49 years	33	94.3	2	5.7	
	50–69 years	26	96.3	1	3.7	
	≥ 70 years	4	100.0	0	0.0	
Marital status	Single/widowed	63	91.3	6	8.7	1.000
	Married	40	93.0	3	7.0	
Occupation	Employed	18	94.7	1	5.3	0.394
	Unemployed	54	93.1	4	6.9	
	Not applicable	22	84.6	4	15.4	
	Unspecified	9	100.0	0	0.0	
Location	City	55	91.7	5	8.3	0.834
	Province	44	91.7	4	8.3	
	Unspecified	4	100.0	0	0.0	
Co-morbidity	Yes	50	94.3	3	5.7	0.495
	No	53	89.8	6	10.2	
Diabetes mellitus	Yes	5	100.0	0	0.0	1.000
	No	98	91.6	9	8.4	
Hypertension	Yes	13	100.0	0	0.0	0.595
	No	90	90.9	9	9.1	
Chronic kidney disease	Yes	4	80.0	1	20.0	0.347
	No	99	92.5	8	7.5	
History of urolithiasis	Yes	8	100.0	0	0.0	1.000
	No	95	91.3	9	8.7	
Malignancy	Yes	1	100.0	0	0.0	1.000
	No	102	91.9	9	8.1	
HIV/AIDS	Yes	10	83.3	2	16.7	0.247
	No	93	93.0	7	7.0	
Steroid use	Yes	9	100.0	0	0.0	1.000
	No	94	91.3	9	8.7	
Systemic lupus erythematosus	Yes	8	100.0	0	0.0	1.000
	No	95	91.3	9	8.7	
Nephrotic syndrome	Yes	1	100.0	0	0.0	1.000
	No	102	91.9	9	8.1	
Cerebrovascular disease	Yes	1	100.0	0	0.0	1.000
	No	102	91.9	9	8.1	
Coronary artery disease	No	103	92.0	9	8.0	No test*
Heart failure	Yes	2	100.0	0	0.0	1.000
	No	101	91.8	9	8.2	
COPD	Yes	1	100.0	0	0.0	1.000
	No	102	91.9	9	8.1	
Bronchial asthma	Yes	2	100.0	0	0.0	1.000
	No	101	91.8	9	8.2	
Spina bifida	No	103	92.0	9	8.0	No test*
RTA Type 1	Yes	1	100.0	0	0.0	1.000
	No	102	91.9	9	8.1	
Previous TB	Yes	14	100.0	0	0.0	0.599
	No	89	90.8	9	9.2	
Other organ involvement of TB	Yes	57	89.1	7	10.9	0.296
	No	46	95.8	2	4.2	
Pulmonary TB	Yes	50	87.7	7	12.3	0.162
	No	53	96.4	2	3.6	
Gastrointestinal TB	Yes	24	85.7	4	14.3	0.224
	No	79	94.0	5	6.0	
Abdominopelvic TB	Yes	11	100.0	0	0.0	0.595
	No	92	91.1	9	8.9	
CNS TB	Yes	6	85.7	1	14.3	0.453
	No	97	92.4	8	7.6	
Bone TB	Yes	3	75.0	1	25.0	0.288
	No	100	92.6	8	7.4	
Cutaneous/Wound TB	Yes	3	100.0	0	0.0	1.000
	No	100	91.7	9	8.3	
TB Adenitis	Yes	7	87.5	1	12.5	0.500
	No	96	92.3	8	7.7	
Ear TB	Yes	1	50.0	1	50.0	0.155
	No	102	92.7	8	7.3	
Psoas TB	Yes	1	50.0	1	50.0	0.155
	No	102	92.7	8	7.3	
Dysuria	Yes	7	100.0	0	0.0	1.000
	No	82	90.1	9	9.9	
Hematuria	Yes	7	77.8	2	22.2	0.192
	No	82	92.1	7	7.9	
Urinary retention	Yes	1	100.0	0	0.0	1.000
	No	88	90.7	9	9.3	
Flank pain	Yes	11	100.0	0	0.0	0.592
	No	78	89.7	9	10.3	
Inguinal pain	Yes	1	100.0	0	0.0	1.000
	No	88	90.7	9	9.3	
Umbilical discharge	Yes	3	100.0	0	0.0	1.000
	No	86	90.5	9	9.5	
Fistula (uretero-cutaneous fistula)	Yes	1	100.0	0	0.0	1.000
	No	88	90.7	9	9.3	
Scrotal discharge	Yes	1	100.0	0	0.0	1.000
	No	88	90.7	9	9.3	
Double J stent reinsertion	Yes	1	100.0	0	0.0	1.000
	No	88	90.7	9	9.3	
Pedal edema	Yes	4	100.0	0	0.0	1.000
	No	85	90.4	9	9.6	
Fever	Yes	7	100.0	0	0.0	1.000
	No	82	90.1	9	9.9	
Weakness	Yes	14	93.3	1	6.7	1.000
	No	75	90.4	8	9.6	
Abdominal pain	Yes	6	75.0	2	25.0	0.157
	No	83	92.2	7	7.8	
Abdominal/pelvic mass on diagnostic	Yes	4	100.0	0	0.0	1.000
	No	85	90.4	9	9.6	
Vaginal bleeding	Yes	2	100.0	0	0.0	1.000
	No	87	90.6	9	9.4	
Difficulty of breathing	Yes	10	83.3	2	16.7	0.303
	No	79	91.9	7	8.1	
Others	Yes	9	81.8	2	18.2	0.266
	No	80	92.0	7	8.0	

Bold values indicate statistically significant differences

*No test was done since all patients were classified under the *No* category

*COPD* chronic obstructive pulmonary disease, *HIV/AIDS* human immunodeficiency virus or acquired immunodeficiency syndrome, *RTA Type 1* renal tubular acidosis Type 1, *TB* tuberculosis, *y* year

**Table 10:** Bivariate analysis: serologic characteristics and mortality

Characteristics	Category	Mortality				*p* value
		Survived		Expired		
		Count	Row %	Count	Row %	
Anemia	Yes	84	90.3	9	9.7	0.353
	No	19	100.0	0	0.0	
Thrombocytopenia	Yes	6	100.0	0	0.0	1.000
	No	97	91.5	9	8.5	
Thrombocytosis	Yes	28	93.3	2	6.7	1.000
	No	75	91.5	7	8.5	
Leukocytosis	Yes	47	100.0	0	0.0	**0.010**
	No	56	86.2	9	13.8	
Leukopenia	Yes	5	83.3	1	16.7	0.402
	No	98	92.5	8	7.5	
Hypoalbuminemia	Yes	54	88.5	7	11.5	0.298
	No	42	95.5	2	4.5	
Renal function impairment	Yes	39	95.1	2	4.9	0.481
	No	63	90.0	7	10.0	
Hyperkalemia	Yes	8	88.9	1	11.1	0.550
	No	93	92.1	8	7.9	
Hypokalemia	Yes	23	95.8	1	4.2	0.681
	No	78	90.7	8	9.3	
Hyponatremia	Yes	48	87.3	7	12.7	0.162
	No	51	96.2	2	3.8	
Hypercalcemia	Yes	19	90.5	2	9.5	1.000
	No	76	91.6	7	8.4	

Bold value indicates statistically significant differences

**Table 11:** Bivariate analysis: urinary characteristics and mortality

Characteristics	Category	Mortality				*p* value
		Survived		Expired		
		Count	Row %	Count	Row %	
Acidic pH	Yes	44	93.6	3	6.4	0.504
	No	50	89.3	6	10.7	
Low specific gravity	Yes	31	96.9	1	3.1	0.268
	No	63	88.7	8	11.3	
Proteinuria	None	32	97.0	1	3.0	0.355
	Trace	12	80.0	3	20.0	
	1+	32	88.9	4	11.1	
	2+	15	93.8	1	6.3	
	3+	3	100.0	0	0.0	
Hematuria	Yes	50	94.3	3	5.7	0.310
	No	44	88.0	6	12.0	
Pyuria	Yes	64	91.4	6	8.6	1.000
	No	30	90.9	3	9.1	
Both hematuria and pyuria	Yes	48	96.0	2	4.0	0.162
	No	46	86.8	7	13.2	
Casts	Yes	15	88.2	2	11.8	0.640
	No	79	91.9	7	8.1	
Crystals	Yes	4	100.0	0	0.0	1.000
	No	90	90.9	9	9.1

**Table 12:** Bivariate analysis: treatments and mortality

Characteristics	Category	Mortality				*p* value
		Survived		Expired		
		Count	Row %	Count	Row %	
Anti-Kochs treatment	Yes	64	90.1	7	9.9	0.482
	No	39	95.1	2	4.9	
Underwent operation	Yes	29	100.0	0	0.0	0.167
	No	74	89.0	9	11.0	
Percutaneous tube nephrostomy	Yes	10	100.0	0	0.0	1.000
	No	93	91.2	9	8.8	
DJS insertion	Yes	11	100.0	0	0.0	0.595
	No	92	91.1	9	8.9	
Transurethral resection of bladder tumor	Yes	1	100.0	0	0.0	1.000
	No	102	91.9	9	8.1	
Bladder mass excision	Yes	3	100.0	0	0.0	1.000
	No	100	91.7	9	8.3	
Aspiration of abscess	Yes	1	100.0	0	0.0	1.000
	No	102	91.9	9	8.1	
Ureteroneocystostomy	Yes	1	100.0	0	0.0	1.000
	No	102	91.9	9	8.1	
Ureterotomy	Yes	1	100.0	0	0.0	1.000
	No	102	91.9	9	8.1	
Pelvolithotomy	Yes	1	100.0	0	0.0	1.000
	No	102	91.9	9	8.1	
Radial nephrolithotomy	Yes	1	100.0	0	0.0	1.000
	No	102	91.9	9	8.1	
Subcapsular nephrectomy	Yes	5	100.0	0	0.0	1.000
	No	98	91.6	9	8.4	
Cytoreductive nephrectomy	Yes	1	100.0	0	0.0	1.000
	No	102	91.9	9	8.1	
Nephrectomy	Yes	2	100.0	0	0.0	1.000
	No	101	91.8	9	8.2

**Table 13:** Bivariate analysis: other outcomes and mortality

Characteristics	Category	Mortality				*p* value
		Survived		Expired		
		Count	Row %	Count	Row %	
Need for renal replacement therapy	Yes	1	100.0	0	0.0	1.000
	No	102	91.9	9	8.1	
Need for pressors	Yes	8	53.3	7	46.7	**<0.001**
	No	95	97.9	2	2.1	

Bold value indicates statistically significant differences

#### Need for pressors

Age, other organ involvement of MTB, gastrointestinal TB, and cutaneous or wound TB, were significantly associated with the need for pressors (p-values of 0.019, 0.035, 0.006, and 0.003, respectively) (Table 14). Other characteristics were not significantly associated with the need for pressors (Tables 14, 15, 16, 17, 18).

**Table 14:** Bivariate analysis: clinical characteristics and need for pressors

Characteristic	Category	Mortality				*p* value
		Survived		Expired		
		Count	Row %	Count	Row %	
Gender	Male	8	15.1	45	84.9	1.000
	Female	10	15.4	55	84.6	
Age	0 months to 1 year	1	100.0	0	0.0	**0.019**
	1–5 years	2	66.7	1	33.3	
	6–10 years	0	0.0	4	100.0	
	11–18 years	4	21.1	15	78.9	
	19–29 years	5	21.7	18	78.3	
	30–49 years	3	8.1	34	91.9	
	50–69 years	2	7.4	25	92.6	
	≥ 70 years	1	25.0	3	75.0	
Marital status	Single/widowed	13	17.6	61	82.4	0.436
	Married	5	11.4	39	88.6	
Occupation	Employed	3	15.0	17	85.0	0.762
	Unemployed	8	13.1	53	86.9	
	Not applicable	6	21.4	22	78.6	
	Unspecified	1	11.1	8	88.9	
Location	City	10	16.1	52	83.9	0.898
	Province	7	13.7	44	86.3	
	Unspecified	1	20.0	4	80.0	
Co-morbidity	Yes	8	14.5	47	85.5	1.000
	No	10	15.9	53	84.1	
Diabetes mellitus	Yes	0	0.0	5	100.0	1.000
	No	18	15.9	95	84.1	
Hypertension	Yes	0	0.0	13	100.0	0.214
	No	18	17.1	87	82.9	
Chronic kidney disease	Yes	2	33.3	4	66.7	0.227
	No	16	14.3	96	85.7	
History of urolithiasis	Yes	2	25.0	6	75.0	0.352
	No	16	14.5	94	85.5	
Malignancy	Yes	0	0.0	1	100.0	1.000
	No	18	15.4	99	84.6	
HIV/AIDS	Yes	2	15.4	11	84.6	1.000
	No	16	15.2	89	84.8	
Steroid use	Yes	3	33.3	6	66.7	0.139
	No	15	13.8	94	86.2	
Systemic lupus erythematosus	Yes	2	25.0	6	75.0	0.352
	No	16	14.5	94	85.5	
Nephrotic syndrome	Yes	1	100.0	0	0.0	0.153
	No	17	14.5	100	85.5	
Cerebrovascular disease	Yes	0	0.0	1	100.0	1.000
	No	18	15.4	99	84.6	
Coronary artery disease	No	18	15.3	100	84.7	No test*
Heart failure	Yes	0	0.0	2	100.0	1.000
	No	18	15.5	98	84.5	
COPD	Yes	0	0.0	1	100.0	1.000
	No	18	15.4	99	84.6	
Bronchial asthma	Yes	0	0.0	2	100.0	1.000
	No	18	15.5	98	84.5	
Spina bifida	No	18	15.3	100	84.7	No test*
RTA Type 1	Yes	0	0.0	1	100.0	1.000
	No	18	15.4	99	84.6	
Previous TB	Yes	0	0.0	14	100.0	0.124
	No	18	17.3	86	82.7	
Other organ involvement of TB	Yes	15	21.4	55	78.6	**0.035**
	No	3	6.3	45	93.8	
Pulmonary TB	Yes	13	21.0	49	79.0	0.079
	No	5	8.9	51	91.1	
Gastrointestinal TB	Yes	10	32.3	21	67.7	**0.006**
	No	8	9.2	79	90.8	
Abdominopelvic TB	Yes	3	23.1	10	76.9	0.417
	No	15	14.3	90	85.7	
CNS TB	Yes	3	37.5	5	62.5	0.102
	No	15	13.6	95	86.4	
Bone TB	Yes	1	25.0	3	75.0	0.489
	No	17	14.9	97	85.1	
Cutaneous/Wound TB	Yes	3	100.0	0	0.0	**0.003**
	No	15	13.0	100	87.0	
TB Adenitis	Yes	1	10.0	9	90.0	1.000
	No	17	15.7	91	84.3	
Ear TB	Yes	1	50.0	1	50.0	0.283
	No	17	14.7	99	85.3	
Psoas TB	Yes	1	50.0	1	50.0	0.283
	No	17	14.7	99	85.3	
Dysuria	Yes	2	28.6	5	71.4	0.603
	No	16	16.7	80	83.3	
Hematuria	Yes	1	11.1	8	88.9	1.000
	No	17	18.1	77	81.9	
Urinary retention	Yes	0	0.0	1	100.0	1.000
	No	18	17.6	84	82.4	
Flank pain	Yes	1	8.3	11	91.7	0.687
	No	17	18.7	74	81.3	
Inguinal pain	Yes	0	0.0	1	100.0	1.000
	No	18	17.6	84	82.4	
Umbilical discharge	Yes	0	0.0	3	100.0	1.000
	No	18	18.0	82	82.0	
Fistula (uretero-cutaneous fistula)	Yes	0	0.0	1	100.0	1.000
	No	18	17.6	84	82.4	
Scrotal discharge	Yes	0	0.0	1	100.0	1.000
	No	18	17.6	84	82.4	
Double J stent reinsertion	Yes	0	0.0	1	100.0	1.000
	No	18	17.6	84	82.4	
Pedal edema	Yes	1	25.0	3	75.0	0.542
	No	17	17.2	82	82.8	
Fever	Yes	0	0.0	7	100.0	0.349
	No	18	18.8	78	81.3	
Weakness	Yes	2	13.3	13	86.7	1.000
	No	16	18.2	72	81.8	
Abdominal pain	Yes	3	27.3	8	72.7	0.402
	No	15	16.3	77	83.7	
Abdominal/pelvic mass on diagnostic	Yes	0	0.0	4	100.0	1.000
	No	18	18.2	81	81.8	
Vaginal bleeding	Yes	0	0.0	2	100.0	1.000
	No	18	17.8	83	82.2	
Difficulty of breathing	Yes	4	30.8	9	69.2	0.235
	No	14	15.6	76	84.4	
Others	Yes	4	36.4	7	63.6	0.098
	No	14	15.2	78	84.8	

Bold values indicate statistically significant differences

*No test was done since all patients were classified under the *No* category

*COPD* chronic obstructive pulmonary disease, *HIV/AIDS* human immunodeficiency virus or acquired immunodeficiency syndrome, *RTA Type 1* renal tubular acidosis Type 1, *TB* tuberculosis, *y* year

**Table 15:** Bivariate analysis: serologic characteristics and need for pressors

Characteristics	Category	Mortality				*p* value
		Survived		Expired		
		Count	Row %	Count	Row %	
Anemia	Yes	16	16.2	83	83.8	0.734
	No	2	10.5	17	89.5	
Thrombocytopenia	Yes	1	16.7	5	83.3	1.000
	No	17	15.2	95	84.8	
Thrombocytosis	Yes	4	13.3	26	86.7	1.000
	No	14	15.9	74	84.1	
Leukocytosis	Yes	8	15.4	44	84.6	1.000
	No	10	15.2	56	84.8	
Leukopenia	Yes	1	16.7	5	83.3	1.000
	No	17	15.2	95	84.8	
Hypoalbuminemia	Yes	14	21.2	52	78.8	0.116
	No	4	8.9	41	91.1	
Renal function impairment	Yes	7	16.7	35	83.3	0.794
	No	11	14.7	64	85.3	
Hyperkalemia	Yes	2	22.2	7	77.8	0.628
	No	16	15.0	91	85.0	
Hypokalemia	Yes	4	16.7	20	83.3	1.000
	No	14	15.2	78	84.8	
Hyponatremia	Yes	12	20.3	47	79.7	0.204
	No	6	10.9	49	89.1	
Hypercalcemia	Yes	4	16.7	20	83.3	1.000
	No	13	15.1	73	84.9

**Table 16:** Bivariate analysis: urinary characteristics and need for pressors

Characteristics	Category	Mortality				*p* value
		Survived		Expired		
		Count	Row %	Count	Row %	
Acidic pH	Yes	9	18.0	41	82.0	0.798
	No	9	15.3	50	84.7	
Low specific gravity	Yes	2	5.9	32	94.1	0.053
	No	16	21.3	59	78.7	
Proteinuria	None	4	11.4	31	88.6	0.727
	Trace	2	13.3	13	86.7	
	1+	7	17.9	32	82.1	
	2+	4	23.5	13	76.5	
	3+	1	33.3	2	66.7	
Hematuria	Yes	8	14.8	46	85.2	0.797
	No	10	18.2	45	81.8	
Pyuria	Yes	11	15.3	61	84.7	0.786
	No	7	18.9	30	81.1	
Both hematuria and pyuria	Yes	7	14.0	43	86.0	0.609
	No	11	18.6	48	81.4	
Casts	Yes	4	22.2	14	77.8	0.493
	No	14	15.4	77	84.6	
Crystals	Yes	0	0.0	4	100.0	1.000
	No	18	17.1	87	82.9

**Table 17:** Bivariate analysis: treatments and need for pressors

Characteristics	Category	Mortality				*p* value
		Survived		Expired		
		Count	Row %	Count	Row %	
Anti-Kochs treatment	Yes	14	18.7	61	81.3	0.196
	No	4	9.3	39	90.7	
Underwent operation	Yes	1	3.3	29	96.7	0.095
	No	17	19.3	71	80.7	
Percutaneous tube nephrostomy	Yes	1	10.0	9	90.0	1.000
	No	17	15.7	91	84.3	
DJS insertion	Yes	0	0.0	12	100.0	0.209
	No	18	17.0	88	83.0	
Transurethral resection of bladder tumor	Yes	0	0.0	1	100.0	1.000
	No	18	15.4	99	84.6	
Bladder mass excision	Yes	0	0.0	3	100.0	1.000
	No	18	15.7	97	84.3	
Aspiration of abscess	Yes	0	0.0	1	100.0	1.000
	No	18	15.4	99	84.6	
Ureteroneocystostomy	Yes	0	0.0	1	100.0	1.000
	No	18	15.4	99	84.6	
Ureterotomy	Yes	0	0.0	1	100.0	1.000
	No	18	15.4	99	84.6	
Pelvolithotomy	Yes	0	0.0	1	100.0	1.000
	No	18	15.4	99	84.6	
Radial nephrolithotomy	Yes	0	0.0	1	100.0	1.000
	No	18	15.4	99	84.6	
Subcapsular nephrectomy	Yes	0	0.0	5	100.0	1.000
	No	18	15.9	95	84.1	
Cytoreductive nephrectomy	Yes	0	0.0	1	100.0	1.000
	No	18	15.4	99	84.6	
Nephrectomy	Yes	0	0.0	2	100.0	1.000
	No	18	15.5	98	84.5

**Table 18:** Bivariate analysis: other outcomes and need for pressors

Characteristics	Category	Need for pressors				*p* value
		Yes		No		
		Count	Row %	Count	Row %	
Need for renal replacement therapy	Yes	0	0.0	1	100.0	1.000
	No	18	15.4	99	84.6	

#### Mean hospital stay

Marital status, steroid use, systemic lupus erythematosus (SLE), other organ involvement of MTB, pulmonary TB, psoas TB, the presence of anemia, leukocytosis, hypoalbuminemia, hyponatremia, hypercalcemia, and anti-Koch’s treatment had a statistically longer mean length of hospital stay compared to those without these characteristics (Tables 19, 20, 22). Other characteristics were not significantly associated with a longer mean length of hospital stay (Tables 19, 20, 21, 22, 23).

**Table 19:** Comparison of mean hospital stay by clinical characteristics

Characteristic	Category	Hospital length of stay (in days)		*p* value
		Mean	SD	
Gender	Male	15.5	16.1	0.914
	Female	13.5	9.5	
Age	0 months to 1 years	23.0		0.444
	1–5 years	35.0	47.9	
	6–10 years	9.5	9.4	
	11–18 years	19.6	16.7	
	19–29 years	13.6	9.5	
	30–49 years	13.2	9.9	
	50–69 years	12.1	7.6	
	≥ 70 years	7.0	4.2	
Marital status	Single/widowed	17.2	14.8	**0.001**
	Married	9.6	6.2	
Occupation	Employed	12.2	11.7	0.506
	Unemployed	13.6	9.0	
	Not applicable	18.4	20.1	
	Unspecified	12.4	6.8	
Location	City	15.3	15.4	0.915
	Province	13.3	9.3	
	Unspecified	15.0	10.8	
Co-morbidity	Yes	15.3	12.4	0.338
	No	13.6	13.3	
Diabetes mellitus	Yes	7.8	4.8	0.143
	No	14.7	13.0	
Hypertension	Yes	13.3	9.4	0.897
	No	14.5	13.3	
Chronic kidney disease	Yes	16.5	9.7	0.333
	No	14.3	13.0	
History of urolithiasis	Yes	9.4	5.1	0.297
	No	14.8	13.2	
Malignancy	Yes	17.0		0.427
	No	14.4	12.9	
HIV/AIDS	Yes	14.5	10.9	0.740
	No	14.4	13.1	
Steroid use	Yes	28.7	18.5	**0.002**
	No	13.2	11.6	
Systemic lupus erythematosus	Yes	23.5	10.9	**0.008**
	No	13.7	12.8	
Nephrotic syndrome	Yes	70.0		0.091
	No	13.9	11.8	
Cerebrovascular disease	Yes	5.0		0.270
	No	14.5	12.9	
Coronary artery disease	Yes			No test*
	No	14.4	12.9	
Heart failure	Yes	19.0	18.4	0.669
	No	14.3	12.8	
COPD	Yes	11.0		0.953
	No	14.4	12.9	
Bronchial asthma	Yes	5.5	2.1	0.150
	No	14.5	12.9	
Spina bifida	Yes			No test*
	No	14.4	12.9	
RTA Type 1	Yes	11.0		0.953
	No	14.4	12.9	
Previous TB	Yes	9.9	5.6	0.204
	No	15.0	13.4	
Other organ involvement of TB	Yes	16.4	14.6	**0.030**
	No	11.5	9.2	
Pulmonary TB	Yes	17.1	15.3	**0.021**
	No	11.4	8.6	
Gastrointestinal TB	Yes	17.7	19.1	0.489
	No	13.2	9.6	
Abdominopelvic TB	Yes	16.0	10.4	0.273
	No	14.2	13.2	
CNS TB	Yes	22.5	27.7	0.309
	No	13.8	11.1	
Bone TB	Yes	34.5	37.8	0.157
	No	13.7	10.9	
Cutaneous/Wound TB	Yes	19.7	7.1	0.161
	No	14.3	13.0	
TB Adenitis	Yes	13.8	12.8	0.642
	No	14.4	12.9	
Ear TB	Yes	51.5	54.4	0.159
	No	13.8	10.9	
Psoas TB	Yes	61.0	41.0	**0.022**
	No	13.6	10.7	

Bold values indicate statistically significant differences

*No test was done since all patients were classified under the *No* category

*COPD* chronic obstructive pulmonary disease, *HIV/AIDS* human immunodeficiency virus or acquired immunodeficiency syndrome, *RTA Type 1* renal tubular acidosis Type 1, *TB* tuberculosis, *y* year

**Table 20:** Comparison of mean hospital stay by serologic characteristics

Characteristic	Category	Hospital length of stay (in days)		*p* value
		Mean	SD	
Anemia	Yes	15.7	13.5	**0.001**
	No	7.6	4.6	
Thrombocytopenia	Yes	14.2	14.3	0.708
	No	14.4	12.8	
Thrombocytosis	Yes	17.9	19.2	0.315
	No	13.2	9.7	
Leukocytosis	Yes	15.6	11.3	**0.027**
	No	13.4	14.0	
Leukopenia	Yes	16.7	14.1	0.690
	No	14.3	12.8	
Hypoalbuminemia	Yes	17.0	15.3	**0.029**
	No	11.3	8.0	
Renal function impairment	Yes	15.5	10.3	0.123
	No	13.9	14.1	
Hyperkalemia	Yes	10.2	5.2	0.411
	No	14.9	13.3	
Hypokalemia	Yes	11.4	6.8	0.373
	No	15.4	14.0	
Hyponatremia	Yes	16.0	13.5	**0.046**
	No	13.1	12.4	
Hypercalcemia	Yes	16.6	7.6	**0.015**
	No	14.3	14.3	

Bold values indicate statistically significant differences

**Table 21:** Comparison of mean hospital stay by urinary characteristics

Characteristic	Category	Hospital length of stay (in days)		*p* value
		Mean	Standard Deviation	
Acidic pH	Yes	14.3	11.8	0.745
	No	14.3	13.5	
Low specific gravity	Yes	13.3	9.9	0.724
	No	14.8	13.8	
Proteinuria	None	14.6	8.0	0.145
	Trace	8.5	5.5	
	1+	15.3	15.9	
	2+	14.4	8.7	
	3+	26.0	38.3	
Hematuria	Yes	12.9	8.2	0.709
	No	15.7	15.8	
Pyuria	Yes	14.0	12.7	0.679
	No	14.9	12.8	
Both hematuria and pyuria	Yes	12.7	8.2	0.609
	No	15.7	15.4	
Casts	Yes	15.6	16.7	0.722
	No	14.1	11.8	
Crystals	Yes	10.3	3.2	0.693
	No	14.5	12.9

**Table 22:** Comparison of mean hospital stay by treatments

Characteristic	Category	Hospital length of stay (in days)		*p* value
		Mean	Standard Deviation	
Anti-Kochs treatment	Yes	17.1	13.9	**<0.001**
	No	9.7	9.1	
Underwent operation	Yes	13.0	9.8	0.666
	No	14.9	13.8	
Percutaneous tube nephrostomy	Yes	16.1	12.0	0.436
	No	14.2	13.0	
DJS insertion	Yes	14.4	9.8	0.762
	No	14.4	13.2	
Transurethral resection of bladder tumor	Yes	6.0		0.370
	No	14.5	12.9	
Bladder mass excision	Yes	5.3	3.1	0.074
	No	14.6	12.9	
Aspiration of abscess	Yes	18.0		0.347
	No	14.4	12.9	
Ureteroneocystostomy	Yes	9.0		0.724
	No	14.4	12.9	
Ureterotomy	Yes	5.0		0.270
	No	14.5	12.9	
Pelvolithotomy	Yes	11.0		0.953
	No	14.4	12.9	
Radial nephrolithotomy	Yes	11.0		0.953
	No	14.4	12.9	
Subcapsular nephrectomy	Yes	11.6	3.6	0.936
	No	14.5	13.1	
Cytoreductive nephrectomy	Yes	7.0		0.481
	No	14.5	12.9	
Nephrectomy	Yes	17.0	12.7	0.602
	No	14.3	12.9	

Bold value indicates statistically significant differences

**Table 23:** Comparison of mean hospital stay by other outcomes

Characteristics	Category	Hospital length of stay (in days)		*p* value
		Mean	Standard Deviation	
Need for renal replacement therapy	Yes	23.0		0.246
	No	14.3	12.9	
Need for pressors	Yes	20.2	24.0	0.790
	No	13.3	9.4	

## Discussion

Genitourinary TB is the second and third most common form of EPTB in countries with high and low TB burden, respectively [9, 19, 25]. According to other registers, however, GUTB is only seen in 1.7–6.5% of the total TB cases reported [26]. In the Philippines, a 5-year retrospective study reported GUTB to have caused 3% of pediatric EPTB cases admitted in a tertiary government hospital [13]. It is important to emphasize that this infection is underdiagnosed in most health care centers, as GUTB remains a diagnostic challenge [9, 19, 25–27].

Diagnosis of GUTB is often delayed due to the insidious nature of the disease, non-specificity of symptoms, poor health-seeking behavior of patients, and lack of clinician awareness [28, 29]. In autopsy studies, only half of patients with renal involvement had symptoms, while only 18% were diagnosed clinically [30]. The four pillars to GUTB diagnosis are bacteriology, pathomorphology, radiology, and provocative test with therapy *ex juvantibus* [6, 19, 31], with culture as the gold standard [25, 29, 32]. In world literature, most cases of GUTB (64.2%) were diagnosed through identification of MTB in the urine, mostly establishing the diagnosis via positive urine culture [9]. Similarly, most retrospective studies in the Asia–Pacific region diagnosed infection based on bacteriologic and histologic findings with consistent clinical history, while a few depended mainly on clinico-radiologic evaluation (Table 24). In our study, the lack of clinical registries of GUTB in our hospital prompted us to instead use laboratory registries for case finding, noting majority of cases being diagnosed based on positivity for urine AFB smear (50.00%). In contrast, one local study observed mycobacterial culture being sent in only 22.2% of urologic cases and in 11.1% of gynecology cases, relying more heavily on clinical, radiographic, and histopathologic assessment [16]. Despite being a recognized tool for diagnosis of GUTB, imaging is particularly useful only during the later stages of the disease when calcifications or cavernous forms have already developed [19, 33]. Nonetheless, this diversity in practice standards is expected in developing countries because of the disproportionate availability of medical facilities and services [34].

**Table 24:** Studies in the Asia–Pacific region involving patients with GUTB

Study (publication year)	Size	Setting (country)	Population	Outcomes	Method
Mishra [33] (2020)	53	Department of Urology, Indira Gandhi Institute of Medical Sciences (India)	Patients with confirmed GUTB	Demographic, clinical presentation Urinary profile, routine blood exams Urine AFB smear test, urine MTB culture Radiological examinations, cystoscopic examination, histopathological examinations	4-year prospective observational case series
Huang [10] (2019)	57	Chang Gung Memorial Hospital-Chiayi (Taiwan)	Patients with diagnosis of GUTB with at least one of the following: positive MTB culture or histologic evidence	Demographics, comorbidities, symptoms and signs Results of mycobacterial smears and cultures, histopathology CBCs, serum biochemistry profile Chest radiography GU tract operations, anti-TB therapy, complications, clinical outcomes	15-year retrospective study
Kim [35] (2018)	56	Severance Hospital, Seoul (South Korea)	Participants older than 18 years diagnosed with GUTB based on presence of any clinical finding plus a positive result for one of the ff: (1) urine AFB, (2) urine MTB culture, (3) urine MTB PCR, or (4) histopathology	Clinical and laboratory data Diagnostic methods, treatment modalities and outcomes	11-year retrospective study
Cao [36] (2017)	419	Peking University First Hospital (China)	All patients with clinical renal TB with microbiologic or histologic confirmation	Demographics, clinical data, complications, treatment Laboratory findings Imaging findings Pathologic features	15-year retrospective study
Krishnamoorthy [8] (2017)	110	Chennai, Tamil Nadu (India)	Patients with either (1) proven GUTB based on urine AFB smear, AFB culture, histopathological evidence of TB, and/or by serological methods; or (2) presumed GUTB who had ≥ 2 consistent features on urological imaging or endoscopic evaluation	Clinical history and examination Serum biochemistry Urine culture Imaging findings	3-year retrospective study
Ye [37] (2016)	193	West China Hospital, Sichuan University (China)	Cases with definite UTB based on results of comprehensive diagnosis, including clinical features, laboratory results (i.e., smear microscopy, MTB culture, real-time PCR, and histological patterns), radiological findings, and response to anti-TB therapy	Demographic data, clinical history, prognosis Radiological findings Selected laboratory results	5-year cross-sectional study
Singh [38] (2013)	117	Urology Department of Institute of Post Graduate Medical Education and Research and SSKM Hospital (India)	All cases clinically diagnosed as GUTB	Clinical presentation Urine AFB smear, urine MTB culture, urine PCR for MTB Radiological and histopathological examinations	13-year retrospective study
Chandra [39] (2012)	25	Himalayan Institute of Medical Sciences, Uttarkhand State (India)	Male patients with histopathologically confirmed GUTB	Occupation, socioeconomic status Clinical history Relevant radiological, laboratory and histopathology findings Treatment	13-year retrospective study
Hsu [40] (2011)	64	National Taiwan University Hospital and Taipei Medical University – Wan Fang Hospital (Taiwan)	All patients with urine culture-confirmed GUTB	Clinical features Laboratory characteristics Treatment outcomes Genotypic characteristics of MTB isolates	12-year retrospective study
Lee [17] (2011)	101	Department of Urology, Hanyang University College of Medicine (Korea)	Patients diagnosed with GUTB based on the presence of one or more positivities in terms of histopathological findings, urine AFB smear, urine MTB culture, and urine PCR for MTB	Yearly proportion, gender, patient distribution according to age, history of TB, and presence of other organ TB Urinalysis findings	10-year retrospective study
Karnjanawanichkul [41] (2010)	35	Prince of Songkla University, Hat Yai, Songkhla (Thailand)	Patients diagnosed with urinary tract TB by demonstration of AFB in urine smear, growth from urine MTB culture, or consistent histopathologic findings	Demographic data, clinical features Laboratory data Chest x-ray, intravenous urography, ultrasonography, or endoscopic findings	10-year retrospective study
Takahashi [42] (2007)	12	Urology clinics of six medical centers, Hokkaido (Japan)	Patients diagnosed with urinary TB based on NAAT or histopathology	Demographic data, clinical features Detection method for MTB Diagnostic findings Treatment, outcomes, and medication-related adverse events	5-year retrospective study
Hsieh [18] (2006)	31	Kaohsiunng Medical University Hospital, Kaohsiung (Taiwan)	Patients diagnosed with GUTB based on microbiological or histological findings plus compatible clinical and roentgenographic findings	Baseline characteristics, underlying diseases, treatment responses, and outcomes	11-year retrospective study
Buccholz [43] (2000)	55	Aga Khan University Hospital (Pakistan)	In-patients with GUTB proven either by urine culture positivity for MTB, or histopathology	Age, sex, concomitant diseases, medical history, symptoms, diagnosis, treatment and follow-up	13-year retrospective study
Ramanathan [34] (1998)	38	Sanjay Gandhi Post Graduate Institute of Medical Sciences, Lucknow (India)	All patients with either: (1) urinary TB based on positive urine or pus cultures for MTB or histopathology, or (2) presumed urinary TB with ≥ 3 consistent features on urological imaging or endoscopy	History and physical examination Serum chemistry Urine culture Chest x-ray and ultrasonography	8-year retrospective study
Dy [16] (1995)	61	Santo Tomas University Hospital (Philippines)	In-patients clinically diagnosed with GUTB	Demographic features Presenting manifestations, history of previous TB Diagnostic modalities (radiographic, bacteriologic, histopathologic) Therapeutic modalities	Case series
Tanchuco [15] (1987)	42	Philippine General Hospital and National Kidney Institute (Philippines)	Patients with discharge diagnosis of urinary tract TB based on the presence of one of the following: positive urine AFB smear, positive urine AFB culture, or consistent histopathologic findings	Clinical and laboratory parameters	6-year retrospective study

*GU* genitourinary, *MTB* Mycobacterium tuberculosis, *NAAT* nucleic acid amplification test, *PTB* pulmonary tuberculosis, *UTB* urinary tuberculosis

Many reviews of the world literature [28, 29] noted kidneys to be the most frequently organ, which is consistent with most studies done in the Asia–Pacific region (Table 25). In contrast, some reports observed the bladder to be the most frequently affected genitourinary organ [38, 40], similar to our investigation. Renal involvement of TB infection can either be a localized urogenital disease or a part of a disseminated infection [6]. In literature, up to 10% of affected individuals have concomitant active pulmonary TB, suggesting hematogenous or lymphatic spread to this highly vascularized organ [28]. The latent period between pulmonary infection and development of clinical GUTB is described to range from 1 to 46 years, averaging around 22 years [29]. Infection may also be acquired hematogenously from the gut [28]. Other genitourinary organs may become affected through ascent or descent of MTB from a source elsewhere in the genitourinary tract, or from contact with the bacilli shed into the urine [26]. They may also get involved from descending spread from the lymphatics [29] or from sexual intercourse [28].

**Table 25:** Studies in the Asia–Pacific region describing the demographic features of patients with GUTB

Study (publication year)	Age	Male-to-female ratio	Demographic	Genitourinary organs involved (n)	Associated comorbidities (%)
Mishra [33] (2020)	Mean, 39.15 ± 12.62 y	1:1.21 (24:29)	Socioeconomic class: lower (88.7%), middle (9.4%), upper (1.9%)	Kidney (33; 18 unilateral, 15 bilateral involvement), ureter (16; 14 lower ureteral stricture, 1 middle ureteral stricture, 1 multiple strictures), bladder (13)	History of PTB (20.8%)
Huang [10] (2019)	Median, 71 years (range, 33–89 years)	1.85:1 (37:20)		Kidneys (8), kidney and ureter (4), epididymis (3), epididymis and testis (3), kidney and prostate (2), prostate (2), ureter (1), ureter and bladder (1), testis (1), epididymis and testis and prostate (1), scrotum and penis (1), uterus and cervix (1)	DM type II (35.1%), chronic renal disease (33.3%), underlying malignancies (hepatocellular, prostate, bladder, cervix, rectum, thyroid gland, lymphoma, and skin) (24.6%), adrenal insufficiency (24.6%), corticosteroid use (21.1%), chronic airway disease (19.3%), liver cirrhosis (17.5%), past history of TB (15.8%), alcoholism (8.8%), and autoimmune disease (3.5%)
Kim [35] (2018)	Mean, 52.8 y	1:1.15 (26:30)		Kidney or ureter (39, 69.6%), bladder (16, 28.6%), epididymis or testis (13, 23.2%), uterus or fallopian tubes (5, 8.9%), prostate (4, 7.1%)	History of TB (42.9%, PTB 37.5%), CVD (28.6%), immunocompromised state (21.4%), pulmonary disease (10.7%), liver disease (7.1%), DM (5.4%), history of gastrectomy (3.6%)
Cao [36] (2017)	Mean, 42.7 ± 13.4 years (range, 12–78 years)	1:1.29 (183:236)	Unemployed (24.6%), farmer (21%), civil servant (15.5%), worker (10.7%), retiree 9.1%), student (4.3%), other occupations (14.6%)	Left kidney (210, 50.1%), right kidney (171, 40.8%), both (38, 9.1%)	History of PTB (20.3%)
Krishnamoorthy [8] (2017)	Mean, 35.4 years (range, 11–67 years)	1.4:1 (65:45)		Kidney (70), ureters (30), bladder (18), testis and epididymis (6), prostate (4), penis (1)	History of PTB (22.7%), gastrointestinal TB (2.7%)
Ye [37] (2016)	Mean, 42.8 ± 14.95 y	1.64:1 (120:73)			Extra-urinary TB (36.3%)
Singh [38] (2013)	Third decade of life (63.2%)	1:1.51 (47:70)		Kidney (76; 56 unilateral, 20 bilateral involvement), ureter (32), bladder (20), prostate (4), scrotal swelling (6)	Past history of PTB (18.9%)
Chandra [39] (2012)	Mean, 37.7 y	NA; only males were included	Location: hilly region of state (68%), non-hilly region of state (32%) Occupation: farmer (56%), laborer (20%), shopkeeper (8%), student (8%), unknown (8%) Socioeconomic status: low (80%)	Urinary bladder (7, 28%), prostate (6, 24%), epididymis (3, 12%), testes (3, 12%), kidney (2, 8%), ureter (2, 8%), scrotum (1, 4%)	Previous history of TB (36%), alcoholism (28%), diabetes (12%)
Hsu [40] (2011)	Mean, 60.3 ± 16.1 y	1.46:1 (38:26)		Bladder (5), ureter (4), kidney (2), kidney/ureter (1), kidney/ureter/bladder (1), epididymis (3), testis/epididymis (2), testis/epididymis/prostate gland (2), epididymis/prostate gland (1), testis (1), prostate gland (1)	57.8% Disseminated TB (48.4%), PTB (43.8%), DM (23.4%), malignancy (14.1%), COPD (14.1%), previous TB (12.5%), CVD (12.5%), receiving steroids (12.5%), ESRD (6.3%), liver cirrhosis (4.7%), alcoholism (4.7%)
Lee [17] (2011)	Mean, 45.57 ± 12.55 years (range, 19–81 years)	1:1.53 (40:61)		Kidney and/or ureter (80.20%), epididymis and/or testis (14.85%), bladder (3.96%), prostate (0.99%)	Past history of PTB (21.8%), intestinal TB (0.99%), spine TB (0.99%)
Karnjanawanichkul [41] (2010)	Mode, 31–40 years (range,10–76)	1.3:1 (20:15)	Occupation: farmer (34.3%), housewife (20.0%), businessperson (14.3%), government service (14.3%), blue-collar worker (14.3%), and student (2.9%)	Kidney (7; 3 bilateral, 3 left, 1 right), ureter (7; 3 bilateral, 1 left, 3 right), bladder (4), testis (3; 1 left, 2 right), kidneys to urethra (2), kidney + bladder (2), ureter + bladder (1), bladder + urethra (1), urethra (1)	Active or past history of PTB (34.3%)
Takahashi [42] (2007)	Median, 68.5 years (range, 40–90 years)	1:1 (6:6)		Kidney (7), bladder (6), ureter (2)	Active PTB (16.7%)
Hsieh [18] (2006)	Mean, M: 54.4 years (range, 32–75 years), F: 61.8 years (range, 31–81 years)	1:1.21 (14:17)			History of PTB (25.8%)
Buccholz [43] (2000)	Mean, 39.9 ± 17.1 years (7–81 years)	3:1 (41:14)		Kidney (28), bladder (15), ureter (13), testes (5), urethra (1)	Active PTB or EPTB on Category I treatment (93%), on Category II (5.4%), on Category III (1.8%), history of EPTB (11%), DM (33%)
Ramanathan [34] (1998)	Mean, 38.8 years	1:3.22 (9:29)			History of PTB (43.2%)
Dy [16] (1995)	Mean, M: 48.4 ± 17.01 years (range, 21–72), F: 43.3 ± 17.58 years (21–78)	1:2.2 (19:42)		Kidneys (50.8%), kidneys + ureter (4.9%), kidneys + ureter + bladder (1.6%), kidneys + prostate (1.6%), pelvis (8.2%), bladder (1.6%), bladder + ureter (1.6%), epididymis (3.3%), epididymis + testis + vas deferens (1.6%), fallopian tube + peritoneum (1.6%)	Active PTB (47.5%), past history of TB (32.8%), hyperuricemia (13.1%), DM (1.6%), HTN (1,6%), DM + HTN (1.6%), hypertensive renal failure (1.6%), liver cirrhosis (1.6%), congestive heart failure (1.6%), myelofibrosis (1.6%), osteoarthritis and prostatic cancer (1.6%), rheumatoid arthritis (1.6%)
Tanchuco [15] (1987)	Mean, 39 years (range, 2 to 64)	1.3:1 24:18			Past history of TB or exposure (28.6%), malnutrition (4.8%), DM (2.4%)

*COPD* chronic obstructive pulmonary disease, *CVD* cardiovascular disease, *DM* diabetes mellitus, *ESRD* end-stage renal disease, *F* female, *HTN* hypertension, *M* male, *NA* not applicable, *PTB* pulmonary tuberculosis

Unilateral organ involvement is commonly shown in retrospective, clinical, and autopsy studies [28, 33, 36, 38, 41]. In our investigation, left laterality was observed in 60% of those with kidney and ureter involvement, consistent with other reports [36, 41]. Renal lesions are initially described to be bilateral attributing to hematogenous spread. They generally undergo a period of cicatrization then enter a latent phase of infection, only reactivating the moment an individual becomes immunocompromised. From a single focus, infection eventually progresses, affecting one kidney while sparing the other. This phenomenon accounts for the greater frequency of unilateral renal TB [9].

### Patient characteristics

Our investigation noted several patients having chronic diseases (e.g., diabetes mellitus, hypertension, chronic kidney disease), immunocompromised conditions (e.g., HIV/AIDS, steroid use), history of tuberculosis, or presence of TB in other organs. Traditionally, risk factors for developing TB include malnutrition, immunosuppression, HIV infection, diabetes, chronic kidney or liver disease, smoking, and low socioeconomic status [25, 28]. Factors considered high-risk for GUTB include past or present TB infection, recurrent or resistant urinary tract infection, and fistulas involving the scrotum, perineum, or lumbar area [5]. Emergence of drug-resistant strains of TB as well as anatomical abnormalities of the urogenital tract from congenital conditions, renal cysts, and urolithiasis also predispose to its development [19]. In our study, a substantial number of patients were single or widowed (61.61%), lived in urban areas (53.57%), and were unemployed (51.79%). In comparison, several studies reported most patients to be living in the hilly region of the state (68%), working as farmers (21–56%) or unemployed (20.0–24.6%), and having low socioeconomic status (80.0–88.7%) [33, 36, 39, 41].

Worldwide, cases encountered between developed and developing countries exhibit different patterns. In developed countries, GUTB mainly affects the elderly, ethnic minorities, and immigrants [6, 9]. On the other hand, patients from developing countries are younger due to higher incidence and severity of TB disease. They present with more specific symptoms and complications which are further exacerbated by delays in diagnosis [9]. Our data demonstrated a mean age of 35.79 ± 18.29 years, with 22.32% belonging to the pediatric population. These findings are consistent with most studies in the region (Table 25). GUTB generally has the propensity to infect both men and women of child-bearing age (20–40 years old), with a mean age of 40.7 years (range, 5–88 years) [25, 29]. We also report a male-to-female ratio of 1:1.15 (52:60). A proper estimate is controversial since there is a lack of controlled epidemiological and clinical studies [28]. While some report more men to be affected than women (2:1) [29], others report women to be affected twice as many as men [28]. Even among past local data, sex distribution was inconsistent [15, 16]. Variation between geographical regions might reflect local TB endemicity or study bias, thereby making accurate epidemiological and clinical data on GUTB difficult to obtain [28].

### Clinical manifestations

GUTB does not present with any specific clinical feature and may in fact be asymptomatic [6]. Up to 50% of cases are incidentally diagnosed when patients undergo work-up for other genitourinary disorders [28]. In those with symptoms, storage symptoms (e.g., urinary frequency, urgency, incontinence, nocturia) were the most common presentation on admission, followed by dysuria, hematuria, and lumbar pain [9, 26, 29]. In our study, we recorded 50.89% of patients to have genitourinary manifestations as their chief complaint, with 21.43% having flank or abdominal pain. Several studies also mentioned abdominal or hypogastric pain to be common (19.6%-42.9%) [15, 16, 35, 41].

We noted 21.43% of patients to have systemic symptoms such as weakness and fever as their initial complaints, while 12.50% manifested with difficulty of breathing. Such findings might be explained by the high rates of TB infection in other organ systems (57.14%), with lungs being the most common extra-genitourinary site (50.89%). In some literature, constitutional symptoms such as fever, weight loss, and night sweats are uncommon and, if present, are indicative of concomitant TB outside the genitourinary system. Some patients may initially present with a myriad of symptoms reflective of other concomitant infections like PTB and hence GUTB symptoms and signs are not always defined by the anatomical site of disease [28]. Moreover, secondary bacterial infections can concurrently occur in up to 50% of patients with GUTB [25, 28]. Our data is consistent with most studies in the Asia–Pacific region, with the exception of those done in South Korea where systemic symptoms are relatively uncommon (3–12.5%) [17, 35]. Systemic manifestations are otherwise present in many reports (28.3–75.4%), with fever being the most cited symptom (29–56.1%) (Table 26). Delays in diagnosis may result in disease progression and severe complications seen at presentation [28].

**Table 26:** Studies in the Asia–Pacific region illustrating the clinical presentation of patients with GUTB

Study (publication year)	Time from symptom onset to diagnosis	Systemic symptoms	Genitourinary manifestations
Mishra [33] (2020)		Constitutional symptoms (28.3%)	Irritative voiding symptoms (69.8%), hematuria (56.6%), flank pain (56.6%), associated renal failure (13.2%), infertility and hematospermia (5.6%), scrotal mass (1.9%)
Huang [10] (2019)	Median, 4 m (range, 0.5–50 m)	75.4% Fever (56.1%), malaise/fatigue (36.8), weight loss (31.6%), night sweats (8.8%)	71.9% Gross hematuria (40.4%), frequency/urgency (33.3%), dysuria (29.8%), flank pain (26.3%)
Kim [35] (2018)		Nonspecific symptoms (fever, anorexia, weight loss, sweating, weakness, peripheral lymphadenopathy) (12.5%)	Urinary frequency or dysuria, urethral pain, or irritable voiding symptoms (55.4%); loin or abdominal pain (42.9%); gross hematuria (33.9%); scrotal pain/mass (19.6%); abscess or fistula (5.4%); vaginal bleeding (3.6%)
Cao [36] (2017)		Constitutional symptoms including weight loss, fever, night sweats, and/or fatigue (38.9%)	Lower urinary tract symptoms including frequency, urgency, and odynuria (65.2%); flank pain (37.9%); gross hematuria (26.3%)
Krishnamoorthy [8] (2017)			Loin pain (27.0%), storage symptoms (25.5%), hematuria (12.0%), stone disease (9.1%), palpable mass (8.2%), scrotal sinus (5.5%), infertility (2.7%), gastrointestinal symptoms (2.7%), urosepsis (1.8%), renal failure (1.8%), calcified kidney (0.9%), urinoma (0.9%)
Ye [37] (2016)		Fever (26.4%), night sweat (13.0%), weight loss (10.9%)	Urinary irritation (61.1%), lumbago (49.2%)
Singh [38] (2013)		32.6%	Irritative voiding symptoms (66.47%), hematuria (47.6%), flank pain (33.8%), recurrent urinary tract symptoms (18.9%), scrotal mass (5.1%), colocutaneous fistula (0.8%), nephrocutaneous fistula (1.8%), associated renal failure (14.7%), infertility or hematospermia (3.4%)
Chandra [39] (2012)		Fever and malaise (32.0%)	Urgency and increased frequency of micturition (56%), lumbar pain (56%), dysuria (52%), hematuria (44%), pyuria (40%), infertility (12%), renal failure (8%), recurrent abscess (8%), scrotal lump (8%), scrotal sinus (4%)
Hsu [40] (2011)	182.0 ± 311.1 d (range, 5 to 1245 d)	51.6% Fever (43.8%), fatigue (37.5%), body weight loss (12.5%)	62.5% Dysuria (31.3%), frequency (31.3%), flank pain (28.1%), hematuria (17.2%), scrotal pain or mass (10.9%)
Lee [17] (2011)		Fever (3.0%)	Frequency (40.6%), hematuria (33.7%), dysuria (16.8%), flank pain (16.8%), scrotal swelling (3%)
Karnjanawanichkul [41] (2010)	< 6 m (65.7%), 6–12 m (17.1%), > 1 years (8.6%), uncertain data (8.6%)		Frequency (48.6%), dysuria (42.9%), hematuria (31.4%), abdominal pain or mass (25.7%), urethral pain (20.0%), retention (14.3%), cutaneous fistula (14.3%), renal failure (5.7%)
Takahashi [42] (2007)	Median duration; between initial symptoms and clinic visit: 120 d (range, 3–360 d); between clinic visit and diagnosis: 14 d (7–150 d)		Chief complaint: frequency (58.3%), hematuria (25.0%), positive nuclear matrix protein 22 on screening test for bladder cancer (8.3%), incidental right renal tumor (8.3%)
Hsieh [18] (2006)	Mean, 2 m (range, 5 d to 18 m)	Fever (29.0%), malaise/fatigue (12.9%), night sweats (3.2%), body weight loss (3.2%)	Frequency/urgency (61.3%), dysuria (58.1%), flank pain (35.5%), gross hematuria (32.2%), scrotal mass/pain (16.1%)
Buccholz [43] (2000)		Fever (36.0%), lassitude (13.0%), weight loss (13.0%)	Dysuria (49%), frequency (40%), flank pain (36%), gross hematuria (31%), urgency (15%), testicular swelling (13%), suprapubic pain (9%), renal colic (1%)
Ramanathan [34] (1998)			Pain (63.6%), hematuria (61.3%), lump (18.2%)
Dy [16] (1995)	Mean, M: 30.4 ± 42.09 m (range, 0.25–180), F: 27.8 ± 35.2 m (0.5–168)	Fever (29.5%), weight loss (18.0%), chills (9.8%), nausea/vomiting (6.6%), anorexia (6.6%)	Dysuria (32.8%), flank pain (27.9%), hematuria (19.6%), hypogastric pain (19.6%), nocturia (19.6%), frequency (18%), edema (14.8%), vaginal spotting (13.1%), costovertebral angle tenderness (11.4%), urgency (9.8%), hesitancy (6.6%)
Tanchuco [15] (1987)		Fever (52.4%), weight loss (26.2%), chills (21.4%), malaise (11.9%), night sweats (2.4%)	Dysuria (71.5%), hematuria (62.0%), flank pain (44.5%), turbid urine (47.6%), frequency (40.5%), hypogastric pain 23.8%), edema (4.8%)

### Hematologic abnormalities

Hematological and biochemical tests are considered non-specific and are instead utilized as adjuncts to GUTB management [28]. In our study, majority of patients presented with anemia (83.04%), while several exhibited leukocytosis (41.96%) and thrombocytosis (26.79%). This is similar to past local data, where anemia and leukocytosis were found in 60.0% and 37.0% of patients, respectively [15]. These estimates are higher than what is recorded in literature, where 15.6–46.9% of patients exhibited anemia and 13.0–25.8% had leukocytosis (Table 27). Thrombocytopenia (26.3%) occurred more frequently in some populations [10]. It is important to note that these studies applied different definitions of hematologic abnormalities, making comparison difficult.

**Table 27:** Studies in the Asia–Pacific region showing serologic and urinary profiles of patients with GUTB

Study (publication year)	Hematologic data	Biochemistry	Urinalysis
Mishra [33] (2020)			Acidic urine (98.1%), sterile pyuria (81.1%), pyuria (69.8%), hematuria (58.4%), alkaline urine (1.9%)
Huang [10] (2019)	Anemia (< 10 g/dL) (28.1%), thrombocytopenia (< 150 × 10^12^/L) (26.3%)	Hypoalbuminemia (< 2.5 g/dL) (40.4%)	Pyuria + hematuria (29.8%), isolated hematuria (> 30/HPF) (17.5%), isolated pyuria (> 20/HPF) (12.3%)
Kim [35] (2018)			Pyuria (> 5 WBCs/HPF) (66.1%), hematuria (> 2 RBCs/HPF) (50.0%), proteinuria (19.6%)
Cao [36] (2017)			Pyuria (> 5 WBCs/HPF) (56.3%), hematuria (> 3 RBCs/HPF) (48.8%)
Ye [37] (2016)	Anemia (15.6%), leukocytosis (13.0%)	Increased BUN (23.3%), increased creatinine (20.2%)	Hematuria (63.2%), proteinuria (45.6%), pyuria (19.2%)
Singh [38] (2013)			Sterile pyuria (62.4%), hematuria (61.5%), proteinuria (57.4%)
Hsu [40] (2011)	Anemia (Hb < 12 g/dL) (46.9%), leukocytosis (WBC > 10,000/µL) (17.2%)	Hypoalbuminemia (albumin < 3.5 g/dL) (37.5%), renal function impairment (Cr > 1.5 mg/dL) (18.8%), liver function impairment (ALT > 40 IU/L) (17.2%)	Pyuria or hematuria (WBC > 10/HPF, × 400; RBC > 5/HPF, × 400) (64.1%), aseptic pyuria (53.1%)
Lee [17] (2011)			Proteinuria (57.4%), hematuria (51.5%), pyuria (42.4%)
Karnjanawanichkul [41] (2010)			Acidic urine with pyuria (80.0%)
Hsieh [18] (2006)	Anemia (Hb < 12 g/dL) (25.8%), leukocytosis (WBC > 10,000/µL) (25.8%), leukopenia (WBC < 4000/µL) (0%)	Poor renal function (Cr > 1.5 mg/dL) (58.1%), poor liver function (ALT > 40 IU/L) (16.1%), hyperkalemia (K^+^ > 5.5 meq/L) (3.2%)	Pyuria + hematuria (51.6%), pyuria (WBC > 10/HPF; 400x) (25.8%), hematuria (RBC > 5/HPF; 400x) (12.9%)
Buccholz [43] (2000)			Pyuria (56%), hematuria (36%), sterile pyuria (6%)
Dy [16] (1995)			Hematuria (37.7%), proteinuria (32.8%), pyuria + bacteriuria (32.8%), sterile pyuria (26.2%), pyuria (8.2%), sterile pyuria + bacteriuria (3.3%), cylindruria (13.1%)
Tanchuco [15] (1987)	Anemia Hb < 12 g/dL (60.0%), leukocytosis (WBC > 10,000/µL) (37.0%)	Creatinine clearance < 30 ml/min (75.0%), elevated BUN (> 0.53 mmol/L) (38.9%), elevated serum Cr (> 176.8 mmol/L) (35.1%)	Alkaline urine (pH ≥ 6) (88.9%), pyuria (WBC ≥ 6/HPF) (82.4%), hematuria (RBC ≥ 6/HPF) (61.8%), albuminuria of 2 + or more (55.8%)

*ALT* alanine aminotransferase, *Cr* serum creatinine, *Hb* hemoglobin, *HPF* high-power field, *RBC* red blood cell, *WBC* white blood cell

TB infection is traditionally known to affect various cell lines. It can cause anemia attributed to four mechanisms: chronic disease, nutritional deficiencies, autoimmune hemolytic anemia, and marrow complications. It is described to commonly affect all subtypes of granulocytes during its course, predominantly affecting neutrophils either quantitatively or qualitatively. It may result in transient neutrophilia or, in extreme cases, leukemoid reaction. TB may also cause leukopenia, especially in females, elderly, or those with recurrent infections. Neutropenia may occur from direct suppression of granulopoiesis by activated T cells [44]. Lymphopenia and lymphocytosis are also commonly reported in active TB [45]. Lastly, TB may result in various platelet abnormalities. Thrombocytosis is frequently reactive in nature and is related to the degree of inflammation. It is mediated by increased levels of endogenous thrombopoietin produced as an acute-phase reactant. Thrombocytopenia, on the other hand, is usually from bone marrow infiltration, disseminated intravascular coagulation, immune thrombocytopenic purpura, thrombotic thrombocytopenic purpura, or drug-induced [44].

### Biochemical abnormalities

Among those with laboratory results, hypoalbuminemia (58.10%) and impairment of renal function (36.94%) were commonly found, similar to those reported in literature, with estimates at 37.5–40.4% and 18.8–75.0%, respectively (Table 27). As plasma creatinine concentration is frequently described to be normal in the setting of unilateral renal involvement, increased levels may indicate bilateral renal involvement or presence of concomitant disorders such as interstitial nephritis or glomerulonephritis [46, 47]. In a review of 8961 cases, 5.7% of patients with GUTB were reported to develop end-stage renal disease (ESRD) [29]. Likewise, one retrospective study in South Korea reported occurrence of ESRD in 7.1% of GUTB patients, identifying acute renal failure and old age as independent risk factors for chronic kidney disease [35].

We also observed several electrolyte abnormalities, specifically hyponatremia (50.93%), hypercalcemia (20.19%), hypokalemia (21.82%), and hyperkalemia (8.18%). Mild hyponatremia has been reported in the setting of active pulmonary or miliary TB, with incidences ranging from 11 to 51% [27, 48]. Most cases are due to syndrome of inappropriate antidiuretic hormone secretion (SIADH), with a third having reset osmostat wherein the plasma sodium stabilizes at lower concentration levels. Mechanisms for such persistent ADH release are yet to be explored, but abnormalities in water handling are demonstrated to resolve following successful treatment of infection [27]. Hyponatremia in TB may also be attributed to adrenal insufficiency. Hyponatremia from this condition is accompanied by hyperkalemia and increased urinary potassium excretion. Cerebral salt wasting is another mechanism for hyponatremia, usually seen in patients with tuberculous meningitis [48]. Lastly, patients with kidney TB may also develop salt-losing nephropathy [49].

Tubulointerstitial nephritis (TIN) is one possible complication of TB which might contribute to the multiple electrolyte abnormalities seen in patients with infection. Several case reports described the development of chronic granulomatous TIN in patients with TB as evidenced by renal biopsy [50–53]. Unlike the other studies, one case series in west London found only 18.7% of those with granulomatous inflammation on renal biopsy to exhibit caseation necrosis [53], whereas another study in France raised the possibility of severe TIN in TB despite the absence of renal granuloma [54]. Although reports on this tubulointerstitial disorder are increasing, mechanisms for its development are poorly understood [55].

Hypercalcemia is another electrolyte abnormality commonly cited in patients with TB. Surveys from different countries show prevalence rates up to 11%-48%, noting that the actual estimates of prevalence are difficult to establish since concurrent serum albumin levels are not consistently reported [48]. Renal or extrarenal TB granulomas accounted for the non-physiologic synthesis of 1,25-dihydroxyvitamin D3 and the ensuing hypercalcemia [56]. Calcification is unusual in the early stages of infection, however, in the advanced stages, nearly all kidneys contains calcification [26].

### Urinary abnormalities

Urinalysis is abnormal in up to 90% of patients with GUTB, with findings ranging from mild changes to extreme pyuria [6, 29, 57]. Pyuria with or without microscopic hematuria is seen in majority of patients, whereas heavy proteinuria and cellular casts are not generally observed [6, 26]. Persistent sterile pyuria, pyuria in an acidic urine without growth on routine culture, or symptomatic UTI unresponsive to standard antibiotics should prompt suspicion of GUTB [6, 25, 28, 58]. Our study showed proteinuria (67.96%) and pyuria (67.96%) to be the most common urinary findings, consistent with those cited in literature (Table 27). Low specific gravity was also found to be common (31.07%), possibly reflecting poor urinary concentrating ability especially in the setting of chronic kidney disease or tubulopathy.

### Radiological findings

Various imaging modalities are used to support the diagnosis of GUTB, with findings dependent upon the extent of disease progression. Despite traditionally being used to suggest, and not to confirm or exclude, the presence of the disease, they are still paramount in management and can still be utilized to confidently diagnose GUTB by those with sufficient experience [59].

Intravenous urogram (IVU) is considered the gold standard for imaging in early renal TB [33, 59]. There might be no abnormalities present during early disease, but there usually appear moderate to severe urinary tract changes once patients become symptomatic [59]. Early findings include infundibular narrowing, calyceal erosion or blunting, and papillary necrosis with associated parenchymal scarring and calcification [27]. GUTB is considered if there is simultaneous involvement of both the upper and the lower urinary tracts, especially the kidney and the bladder [27, 29]. Later stages of the disease may demonstrate extensive cavitation, mass lesions, calyceal distortion, cortical scarring, calcification, autonephrectomy, perinephric abscess, fistula formation, ureteral strictures, and bladder fibrosis [25, 59]. In our study, both patients who underwent intravenous pyelography showed advanced findings of extensive stone formation and non-functioning kidney.

Triphasic computed tomography (CT) scan remains the mainstay imaging technique for cross-sectional imaging in GUTB [6]. It is the most sensitive modality to detect calcification, and is superior to IVU in detecting multiple small urothelial lesions [25, 60]. Findings suggestive of GUTB include the presence of lesions in other organs beyond the urinary tract, such as liver, lymph nodes, and vertebrae [29], as seen in our study.

Although IVU and CT scan are reported to be the more frequently used imaging modalities in GUTB [29], some investigations still heavily rely on ultrasonography [18, 36]. Ultrasonography may only give indirect evidence of GUTB, but it avoids exposure to ionizing radiation and can be conveniently used to guide fine needle aspiration biopsies [5, 25, 59]. In literature, two patterns of GUTB have been described: (1) the infiltrative pattern showing increased echogenicity from calcifications, infected debris, or abscesses; and (2) hydronephrosis or pyonephrosis, involving calyceal dilatation and a small renal pelvis. Another distinguishing feature for GUTB is the visualization of multiple abnormalities in different disease stages with various organ involvement [59]. All these changes were observed in our investigation.

### Treatment

Medical treatment of GUTB should be initiated promptly when clinical, laboratory, and radiological findings suggest a presumptive diagnosis, even prior to the release of microbiologic and histopathologic results [26, 47]. Pharmacologic therapy for drug-sensitive TB consists of an intensive phase of quadruple therapy with first-line anti-TB agents (e.g., isoniazid, rifampicin, pyrazinamide and ethambutol) for 2 months, followed by a continuation phase with two drugs (e.g., isoniazid, rifampicin) for 4 months [28]. In our study, over sixty percent (63.39%) of patients were started on anti-TB therapy during admission, reflecting diverse in-hospital practices wherein several cases were discharged upon resolution of their reasons for encounter, with eventual treatment initiated on an out-patient basis after microbiologic and histopathologic confirmation.

Around half (54.9%) of patients with GUTB require surgery [28, 47]. Indications for surgery include diversion of urologic obstruction, drainage of abscesses, nephrectomy of non-functioning kidneys, reconstruction of affected ureters, and dilation of contracted urinary bladder [26, 28]. A quarter (25.89%) of patients in our study underwent an operation, lower than that observed in a previous local investigation (52%) [15]. This difference might be attributed to the higher proportion of younger individuals involved in our study, whose illness duration may not be long enough to develop complications that would warrant surgical therapy. This finding might signify a changing pattern of disease in the country.

### Short-term outcomes

Deaths from tuberculosis are higher in developing (2–3 million deaths per year) than developed countries (40 thousand deaths per year) [9]. In GUTB, mortality rates vary (1.2–28.1%) [10], with most patients succumbing from disseminated infection [15]. In our center, all-cause in-hospital mortality occurred in 8.04% of the included patients. Age, leukocytosis, and the need for pressors were all significantly associated with mortality (*p* values of <0.001, 0.010, and <0.001, respectively). Several researches in Taiwan investigated risk factors for mortality in patients with GUTB. Fever was shown to be positively correlated with mortality (OR = 42.716; 95% CI 1.032–1767.569; *p* = 0.048), possibly attributed to its high prevalence in elderly patients, those with multiple co-morbidities, and those who had delays in diagnosis [10]. Other poor prognostic factors included genitourinary tract surgery (OR = 0.000; 95% CI 0.000–0.255; *p* = 0.020) [10], age older than 65 years old (HR = 4.03; 95% CI 1.27–12.76; *p* = 0.02), cardiovascular disease (HR = 5.96; 95% CI 1.98–17.92; *p* = 0.001), steroid use (HR = 10.16; 95% CI 2.27–45.47; *p* = 0.02), and no treatment (HR = 4.81; 95% CI 1.12–20.67; *p* = 0.04) [40].

It was observed that patients with longer mean lengths of hospital stay did not necessarily develop unfavorable hospital outcomes. Instead, prolonged admission duration seemed to have been influenced by management of co-morbidities (e.g., SLE, pulmonary TB, psoas TB) and correction of various clinically significant laboratory abnormalities (e.g., anemia, leukocytosis, hyponatremia, and hypercalcemia). Awaiting results of TB workups during admission for diagnosis of equivocal cases and for subsequent initiation of definitive therapy might have also contributed since anti-Koch’s treatment had a statistically longer mean length of hospital stay (*p* <0.001). These findings were reflective of the varying practices in our center, with many cases undergoing surgery for prompt treatment of symptoms and having medical therapy be initiated on an out-patient basis depending on the results of cultures and histopathology.

## Limitations

Our study had several limitations. Its retrospective design which was affected by institutional limitations in recordkeeping made data collection restricted, as evidenced by a low chart retrieval rate (57.89%, 132/228). Given the absence of a unified clinical registry and data recording, our case finding was laboratory-based and was subject to selection bias, thereby rendering our results nongeneralizable to a broader patient population. It also proved to be a major barrier preventing inclusion of predictive modeling of study outcomes. Despite these limitations, our research is the largest study in the country to date, involving more accurate diagnosis of GUTB compared to past local studies since all reviewed cases had bacteriologic or histopathologic evidence of infection. It also involved more specific definitions of serologic findings adjusted per age and sex.

## Conclusion

Our investigation observed a high prevalence rate of serologic and urinary abnormalities among admitted GUTB patients in the study. Apart from the commonly cited abnormalities in literature, multiple electrolyte abnormalities and urinary concentration defects were observed in many cases, possibly indicating tubulointerstitial involvement. Accordingly, we recommend future research be done on such complication to determine its correlation with disease activity and to possibly help with the diagnosis of infection particularly in low-resource settings. Mortality rate was also noted to be high among admitted patients with GUTB. Age, leukocytosis, and need for pressors were significantly associated with mortality. However, further research is recommended to explore predictive modeling.

## Data Availability

The datasets used in this study are available from the corresponding author upon reasonable request.

## References

[1] World Health Organization: Global tuberculosis report 2019. 2019.

[2] Mehta JB, Dutt A, Harvill L, Mathews KM (1991). Epidemiology of extrapulmonary tuberculosis: a comparative analysis with pre-AIDS era. Chest.

[3] Das P, Ahuja A, Gupta SD (2008). Incidence, etiopathogenesis and pathological aspects of genitourinary tuberculosis in India: a journey revisited. Indian J Urol.

[4] Noertjojo K, Tam CM, Chan SL, Chan-Yeung MMW (2002). Extra-pulmonary and pulmonary tuberculosis in Hong Kong. Int J Tuberc Lung Dis.

[5] Kulchavenya E (2014). Urogenital tuberculosis: definition and classification. Ther Adv Infect Dis.

[6] Kulchavenya E, Yasuda M, Grabe M, Bjerklund Johansen TE, Wagenlehner FME, Matsumoto T, Cho YH, Krieger JN, Shoskes D, Naber KG (2019). Epidemiology, classification, clinical features, and diagnostics of urogenital tuberculosis. Urogenital infections and inflammations.

[7] Wildbolz H (1937). Ueber urogenical tuberkulose. Schweiz Med Wochenschr.

[8] Krishnamoorthy S, Palaniyandi V, Kumaresan N, Govindaraju S, Rajasekaran J, Murugappan I (2017). Aspects of evolving genito urinary tuberculosis: a profile of genito urinary tuberculosis (GUTB) in 110 patients. J Clin Diagn Res.

[9] Figueiredo AA, Lucon AM, Junior RF, Srougi M (2008). Epidemiology of urogenital tuberculosis worldwide. Int J Urol.

[10] Huang TY, Hung CH, Hsu WH, Peng KT, Hung MS, Lai LJ (2019). Genitourinary tuberculosis in Taiwan: a 15-year experience at a teaching hospital. J Microbiol Immunol Infect.

[11] Porter MF (1894). Uro-genital tuberculosis in the male. Ann Surg.

[12] Lansang MAD, Alejandria MM, Law I, Juban NR, Amarillo MLE, Sison OT (2021). High TB burden and low notification rates in the Philippines: the 2016 national TB prevalence survey. PLoS ONE.

[13] Santos R (2013). Clinical profile of extrapulmonary tuberculosis cases admitted and diagnosed in a tertiary government hospital from January 2006 to June 2010. Pediatr Infect Dis Soc Philippines J.

[14] Lim RG, Caro HP, Dominguez-Mejia AE, Yuhico MJ (1996). The clinical profile of patients with sterile pyuria and recurrent urinary tract infection. Phillippine J Intern Med.

[15] Tanchuco JQ, Lazo PO, King B (1987). Urinary tract tuberculosis: local experience. Philippine J Nephrol.

[16] Dy EER, Rosales LN, Lim EU (1995). Genitourinary tuberculosis at the Santo Tomas University Hospital. Phillippine J Intern Med.

[17] Lee JY, Park HY, Park SY, Lee SW, Moon HS, Kim YT (2011). Clinical characteristics of genitourinary tuberculosis during a recent 10-year period in one center. Korean J Urol.

[18] Hsieh HC, Lu PL, Chen YH, Chen TC, Tsai JJ, Chang K (2006). Genitourinary tuberculosis in a medical center in southern Taiwan: an eleven-year experience. J Microbiol Immunol Infect.

[19] Kulchavenya E (2013). Best practice in the diagnosis and management of urogenital tuberculosis. Ther Adv Urol.

[20] World Health Organization (2011). Haemoglobin concentrations for the diagnosis of anaemia and assessment of severity.

[21] Lo SF, Kliegman R, St Geme JW, Blum NJ, Tasker RC, Shah SS, Wilson KM (2020). Reference intervals for laboratory tests and procedures. Nelson textbook of pediatrics.

[22] Longo DL, Fauci AS, Kasper DL, Hauser SL, Jameson J, Loscalzo J (2018). Harrison's principles of internal medicine.

[23] Berliner N. Approach to the adult with unexplained neutropenia. Uptodate, 2018. Accessed 3 Feb 2020.

[24] Skorecki K, Chertow GM, Marsden PA, Brenner BM, Rector FC (2016). Brenner & Rector’s the kidney.

[25] Abbara A, Davidson RN (2011). Etiology and management of genitourinary tuberculosis. Nat Rev Urol.

[26] Amaya-Tapia G, Aguirre-Avalos G (2018). Urinary tract tuberculosis. IntechOpen.

[27] Venyo AK, Venyo LK, Maloney DJL, Khan AN (2015). Tuberculosis of the kidney and the genitourinary tract: a review of the literature. Hamdan Med J.

[28] Muneer A, Macrae B, Krishnamoorthy S, Zumla A (2019). Urogenital tuberculosis: epidemiology, pathogenesis and clinical features. Nat Rev Urol.

[29] Figueiredo AA, Lucon AML (2008). Urogenital tuberculosis: update and review of 8961 cases from the world literature. Rev Urol.

[30] Medlar EM, Spain DM, Holliday RW (1949). Post-mortem compared with clinical diagnosis of genitourinary tuberculosis in adult males. J Urol.

[31] Lewinsohn DM, Leonard MK, LoBue PA, Cohn DL, Daley CL, Desmond E (2017). Official American Thoracic Society/Infectious Diseases Society of America/Centers for Disease Control and Prevention clinical practice guidelines: diagnosis of tuberculosis in adults and children. Clin Infect Dis.

[32] Mehta PK, Kamra E (2020). Recent trends in diagnosis of urogenital tuberculosis. Future Microbiol.

[33] Mishra KG, Ahmad A, Singh G, Tiwari R (2020). Current status of genitourinary tuberculosis: presentation, diagnostic approach and management—single centre experience at IGIMS (Ptana, Bihar, India). Indian J Surg.

[34] Ramanathan R, Kumar A, Kapoor R, Bhandari M (1998). Relief of urinary tract obstruction in tuberculosis to improve renal function. Analysis of predictive factors. Br J Urol.

[35] Kim EJ, Lee W, Jeong WY, Choi H, Jung IY, Ahn JY (2018). Chronic kidney disease with genitourinary tuberculosis: old disease but ongoing complication. BMC Nephrol.

[36] Cao Y, Fan Y, Chen Y, Zhao Z, Song Y, Shen C (2017). Gross hematuria is more common in male and older patients with renal tuberculosis in China: a single-center 15-year clinical experience. Urol Int.

[37] Ye Y, Hu X, Shi Y, Zhou J, Zhou Y, Song X (2016). Clinical features and drug-resistance profile of urinary tuberculosis in south-western China. Medicine.

[38] Singh JP, Priyadarshi V, Kundu AK, Vijay MK, Bera MK, Pal DK (2013). Genito-urinary tuberculosis revisited: 13 years’ experience of a single centre. Indian J Tuberc.

[39] Chandra S, Chandra H, Chauhan N, Gaur DS, Gupta H, Pathank VP (2012). Male genitourinary tuberculosis: 13 years experience at a tertiary care center in India. Southeast Asian J Trop Med Public Health.

[40] Hsu H-L, Lai C-C, Yu M-C, Yu F-L, Lee J-C, Chou C-H (2011). Clinical and microbiological characteristics of urine culture-confirmed genitourinary tuberculosis at medical centers in Taiwan from 1995 to 2007. Eur J Clin Microbiol Infect Dis.

[41] Karnjanawanichkul W, Pripatnanont C (2010). Tuberculosis of the urinary tract in southern Thailand. J Med Assoc Thai.

[42] Takahasi S, Takeyama K, Kunishima Y, Hashimoto K, Miyamoto S, Ichihara K (2007). Current survey of urinary tuberculosis in Hokkaido, Japan. J Infect Chemother.

[43] Buccholz NP, Haque R, Salahuddin S (2000). Genitourinary tuberculosis: a profile of 55 in-patients. J Pak Med Assoc.

[44] Balepur SS, Schlossberg D. Hematologic complications of tuberculosis. Microbiol Spectr. 2016. 10.1128/microbiolspec.TNMI7-0004-2016. 10.1128/microbiolspec.TNMI7-0004-2016 28084210

[45] Oyer RA, Schlossberg D, Schlossberg D (1994). Hematologic changes in tuberculosis. Tuberculosis.

[46] Visweswaran RK, Pais VM, Dionne-Odom J. Urogenital tuberculosis. UpToDate, 2019. Accessed 9 Nov 2020.

[47] Figueiredo AA, Lucon AM, Srougi M (2017). Urogenital tuberculosis. Microbiol Spectr.

[48] Vinnard C, Blumberg EA (2017). Endocrine and metabolic aspects of tuberculosis. Microbiol Spectr.

[49] Murphy FD, Settimi AL, Kozokoff NJ (1953). Renal disease with the salt losing syndrome: a report of four cases of so-called “salt losing nephritis”. Ann Intern Med.

[50] Mallison WJ, Fuller RW, Levison DA, Baker LR, Cattell WR (1981). Diffuse interstitial renal tuberculosis: an unusual cause of renal failure. Q J Med.

[51] El-Reshaid KA, Madda JP, Al-Saleh MA (2001). A case of progressive tubulo-interstitial nephritis due to culture-negative renal tuberculosis. Saudi J Kidney Dis Transpl.

[52] Sampathkumar K, Sooraj YS, Mahaldar AR, Ramakrishnan M, Rjappannair A, Nalumakkal SV (2009). Granulomatous interstitial nephritis due to tuberculosis: a rare presentation. Saudi J Kidney Dis Transpl.

[53] Chapagain A, Dobbie H, Sheaff M, Yaqoob M (2011). Presentation, diagnosis, and treatment outcome of tuberculous-mediated tubulointerstitial nephritis. Kidney Int.

[54] Delafosse M, Teuma C, Miailhes P, Nouvier M, Rabeyrin M, Fouque D (2018). Severe tubulointerstitial nephritis: tracking tuberculosis even in the absence of renal granuloma. Clin Kidney J.

[55] Eastwood JB, Corbishley CM, Grange JM (2011). Tuberculosis and tubulointerstitial nephritis: an intriguing puzzle. Kidney Int.

[56] Pouchot J, Dreyfuss D, Gardin JP, Mier L, Remy P, Esdaile JM (1993). Ectopic production of 1,25-dihydroxyvitamin D3 in tuberculosis. Nephrol Dial Transpl.

[57] Daher EDF, da Silva GB, Barros EJG (2013). Review: renal tuberculosis in the modern era. Am J Trop Med Hyg.

[58] American Thoracic Society (2000). Diagnostic standards and classification of tuberculosis in adults and children. Am J Respir Crit Care Med.

[59] Merchant S, Bharati A, Merchant N (2013). Tuberculosis of the genitourinary system: urinary tract tuberculosis: renal tuberculosis—part I. Indian J Radiol Imaging.

[60] Merchant S, Bharati A, Merchant N (2013). Tuberculosis of the genitourinary system: urinary tract tuberculosis: renal tuberculosis—part II. Indian J Radiol Imaging.

